# EPPS rescues hippocampus-dependent cognitive deficits in APP/PS1 mice by disaggregation of amyloid-β oligomers and plaques

**DOI:** 10.1038/ncomms9997

**Published:** 2015-12-08

**Authors:** Hye Yun Kim, Hyunjin Vincent Kim, Seonmi Jo, C. Justin Lee, Seon Young Choi, Dong Jin Kim, YoungSoo Kim

**Affiliations:** 1 Center for Neuro-Medicine, Brain Science Institute, Korea Institute of Science and Technology (KIST), Hwarangno 14-gil 5, Seongbuk-gu, Seoul 136-791, Republic of Korea; 2 Biological Chemistry Program, Korea University of Science and Technology (UST), 217 Gajungro Yuseong-gu, Daegeon 305-806, Republic of Korea; 3 Research Institute, GoshenBiotech Inc., 78, Sure-ro 640beon-gil, Wabu-eup, Namyangju-si, Gyeonggi-do 472-905, Republic of Korea; 4 Center for Neuroscience, Brain Science Institute, Korea Institute of Science and Technology (KIST), Hwarangno 14-gil 5, Seongbuk-gu, Seoul 136-791, Republic of Korea

## Abstract

Alzheimer's disease (AD) is characterized by the transition of amyloid-β (Aβ) monomers into toxic oligomers and plaques. Given that Aβ abnormality typically precedes the development of clinical symptoms, an agent capable of disaggregating existing Aβ aggregates may be advantageous. Here we report that a small molecule, 4-(2-hydroxyethyl)-1-piperazinepropanesulphonic acid (EPPS), binds to Aβ aggregates and converts them into monomers. The oral administration of EPPS substantially reduces hippocampus-dependent behavioural deficits, brain Aβ oligomer and plaque deposits, glial γ-aminobutyric acid (GABA) release and brain inflammation in an Aβ-overexpressing, APP/PS1 transgenic mouse model when initiated after the development of severe AD-like phenotypes. The ability of EPPS to rescue Aβ aggregation and behavioural deficits provides strong support for the view that the accumulation of Aβ is an important mechanism underlying AD.

## Abstract (web summary)

Amyloid-beta deposits are a pathological hallmark of Alzheimer's disease, and have previously been targeted in immunisation therapies. Here, the authors show that oral administration of the small molecule EPPS reduces Aß plaque and oligomer load in APP/PS1 mice and improves learning and memory performance.

## Graphical abstract

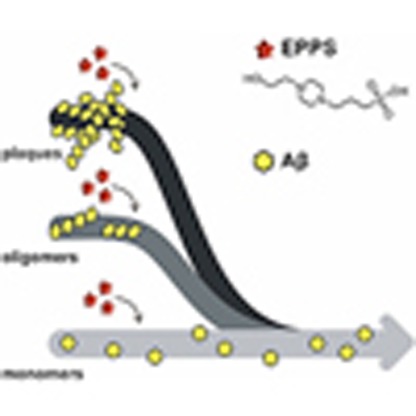

During Alzheimer's disease (AD) pathogenesis, amyloid-β (Aβ) monomers aberrantly aggregate into toxic oligomers, fibrils and eventually plaques. The concentration of misfolded Aβ species highly correlates with the severity of neurotoxicity and inflammation that leads to neurodegeneration in AD^1^,^2^,^3^. Accordingly, substantial efforts have been devoted to reducing Aβ levels, including methods to prevent the production and aggregation of Aβ^4^,^5^,^6^,^7^. Although these approaches effectively prevent the *de novo* formation of Aβ aggregates, existing Aβ oligomers and plaques will still remain in the patient's brain^8^,^9^,^10^. Thus, the desirable effects of Aβ inhibitors may be expected when administered before a patient develops toxic Aβ deposits^5^,^6^,^7^. However, in AD patients with mild-to-moderate symptoms, anti-amyloidogenic agents have not yielded expected outcomes, which may be due to the incomplete removal of pre-existing Aβ aggregates^11^. As Aβ typically begins to aggregate long before the onset of AD symptoms, interventions specifically aimed at disaggregating existing plaques and oligomers may constitute a useful approach to AD treatment, perhaps in parallel with agents aimed at inhibiting aggregate formation^8^,^9^,^10^,^11^,^12^.

## Results

### EPPS reduces Aβ-aggregate-induced memory deficits in mice

**Figure 1:**
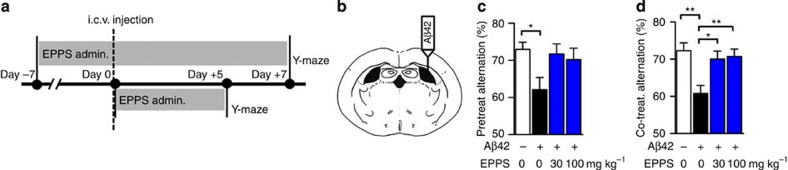
EPPS ameliorates Aβ-induced memory deficits in mice. (**a**) Time course of the experiments. (**b**) Intracerebroventricular (i.c.v.) injection site brain schematic diagram. (**c**) Pretreated effects of EPPS on Aβ-aggregate-induced memory deficits observed by the % alternation on the Y-maze. EPPS, 0 (*n*=10), 30 (*n*=9) or 100 mg kg^−1^ per day (*n*=10), was orally given to 8.5-week-old ICR male mice for 1 week; then, vehicle (10% DMSO in PBS, *n*=10) or Aβ aggregates (50 pmol per 10% DMSO in PBS; Supplementary Fig. 1A) were injected into the intracerebroventricular region (*P*=0.022). (**d**) Co-treated effects of EPPS on Aβ-aggregate-induced memory deficits observed by the % alternation on the Y-maze. Male, 8.5-week-old ICR mice received an injection of vehicle (*n*=9) or Aβ aggregates into the intracerebroventricular region, and then EPPS, 0 (*n*=10), 30 (*n*=10) or 100 mg kg^−1^ per day (*n*=10), was orally given to these mice for 5 days. From the top, *P*=0.003, 0.006, 0.015. The error bars represent the s.e.m. One-way analysis of variance followed by Bonferroni's *post-hoc* comparisons tests were performed in all statistical analyses. (*P<0.05, **P<0.01, ***P<0.001; other comparisons were not significant).

Previously, we reported a series of small ionic molecules that could accelerate the formation of Aβ aggregates *in vitro*. Unexpectedly, in addition to the compounds facilitating Aβ aggregation, we identified six small molecules that inhibited the formation of Aβ oligomers and fibrils^13^. In the current study, we tested whether these molecules could affect AD-like cognitive impairments of rodents. For this purpose, we induced memory deficits in 8.5-week-old Imprinting Control Region (ICR) mice (male, *n*=9–10 per group) by injecting Aβ42 aggregates (Fig. 1a,b) into the intracerebroventricular region^14^. This Aβ-infusion model allowed us to control the onset of abnormal Aβ deposition before or after the administration of our compounds. Among the six orally administered molecules, only 4-(2-hydroxyethyl)-1-piperazinepropanesulphonic acid (EPPS) ameliorated AD-like phenotypes in our mouse model. To assess the cognitive changes of Aβ-infusion mice, we performed Y-maze tests and observed alternations of short-term spatial working memory. To examine the prophylactic efficacy of EPPS in this model (pretreatment), EPPS was orally administered for 7 days (30 or 100 mg kg^−1^ per day) to 8.5-week-old ICR mice (*n*=9–10 per group) via drinking water, followed by the intracerebroventricular injection of Aβ aggregates (Supplementary Fig. 1A) and an additional 7-day oral administration of EPPS. In the Y-maze test, EPPS pre-administration blocked the development of Aβ-induced memory deficits (Fig. 1c). To assess prompt efficacy (co-treatment) of EPPS, we orally administered EPPS to 8.5-week-old ICR mice (*n*=9–10 per group) for 5 days (30 or 100 mg kg^−1^ per day) via drinking water subsequent to the intracerebroventricular injection of Aβ aggregates (Supplementary Fig. 1A). We observed substantial rescue of working memory deficits in Aβ-infused mice by EPPS treatment (treatment effects: 30 mg kg^−1^ per day, *P*=0.015; 100 mg kg^−1^ per day, *P*=0.006; Fig. 1d). Collectively, these results imply that EPPS not only inhibits Aβ aggregation but also mediates Aβ-induced cognitive impairments.

### EPPS is orally safe and penetrates the blood–brain barrier

Before conducting further *in vivo* studies of therapeutic potentials, we measured the toxicity and pharmacokinetics profiles of EPPS. Toxicity and pharmacokinetics are crucial features of AD therapeutics, as long-term treatment is often required. To examine whether EPPS elicits toxic effects when orally administered, we included EPPS in drinking water for wild-type (WT) mice (4-week-old, male, *n*=6 per group) to determine the half-maximal lethal dosage (LD_50_). Lethal toxicity was measured based on mortality, changes in body weight and hair loss observed over a 2-month period. The oral administration of EPPS did not cause any toxicity up to 2,000 mg kg^−1^ per day of administration (LD_50_>2,000 mg kg^−1^ per day). To investigate the ability of EPPS to penetrate the blood–brain barrier, we performed pharmacokinetics profiling in Sprague–Dawley (SD) rats (male, *n*=9 per group; Supplementary Table 1). We orally administered EPPS (0, 10 or 100 mg kg^−1^ per day) via drinking water for 7 consecutive days^5^. During the administration, we did not observe any obvious abnormal behaviours or physical changes. Blood (500 μl) and brain samples were collected at 24, 72, 120 and 168 hours. As the structure of EPPS lacks a chromophore detectable in high-performance liquid chromatography, concentrations of EPPS in the plasma and brain samples were determined using mass spectrometry. The chromatographic conditions showed that the blank plasma or brain homogenate had no interference in the EPPS determination. We found that the brain concentration of EPPS (10 mg kg^−1^ per day) reached its plateau (7.52 ng g^−1^) within 72 hours and sustained a brain/plasma level of 2.04 ml g^−1^ (Supplementary Table 2). At a dose of 100 mg kg^−1^ per day, we observed that the EPPS brain/plasma level (0.342 ml g^−1^) was relatively lower than that of rats with 10 mg kg^−1^ per day administration of EPPS, despite the higher brain concentration. Collectively, orally administered EPPS lacked evident toxicity after daily 2,000 mg kg^−1^ per day dosing for 2 months and maximal brain/plasma levels have been achieved at doses lower than 100 mg kg^−1^ per day. Based on these results, we decided to orally administer 10–100 mg kg^−1^ per day EPPS dosages to mice for the *in vivo* experiments, including behavioural tests and brain analyses.

### Orally administered EPPS rescues cognitive deficits in APP/PS1 mice

**Figure 2:**
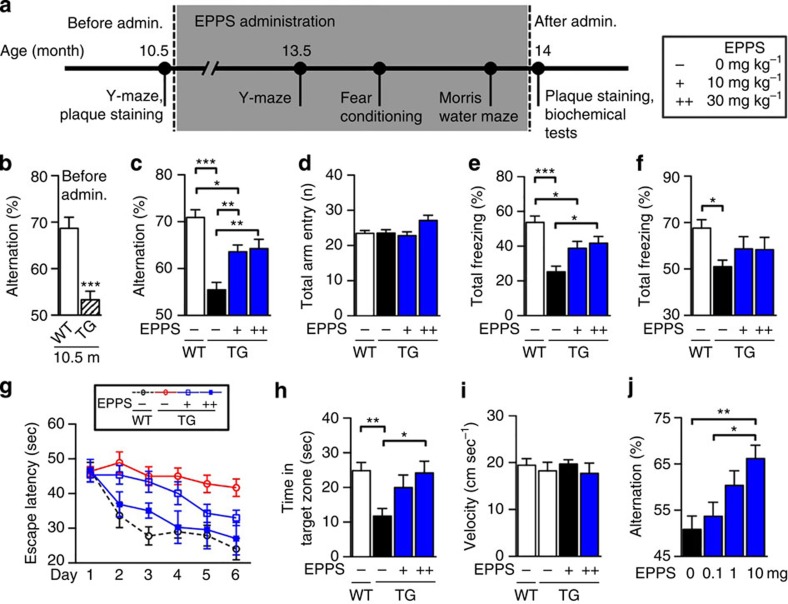
EPPS rescues hippocampus-dependent cognitive deficits. (**a**) Time course of behavioural tests. EPPS, 0 (TG(**−**), male, *n*=15), 10 (TG(**+**), male, *n*=11) or 30 mg kg^−1^ per day (TG(**++**), male, *n*=8), was orally given to 10.5-month-old APP/PS1 mice for 3.5 months and their behavioural changes were compared with age-matched WT mice (WT(**−**), male, *n*=16). (**b**) Pre-EPPS treatment evaluation of cognitive deficits; 10.5-month-old WT mice (male, *n*=18) and age-matched APP/PS1 TG mice (male, *n*=13) were used in Y-maze tests, to obtain the % alternation before the administration of EPPS. The data indicated cognitive deficits in 10.5-month-old APP/PS1 mice (*P*<0.0001). (**c**–**i**) Y-maze, fear-conditioning and Morris water maze tests on 14-month-old APP/PS1 mice after EPPS administration for 3.5 months total. (**c**) Per cent alternation on Y-maze. From the top, *P*=0.000, 0.020, 0.008, 0.010. (**d**) Total entry number into each arm of the Y-maze test. (**e**) Per cent total freezing from contextual fear conditioning. From the top, *P*=0.000, 0.036, 0.038. (**f**) Per cent total freezing in the cued task, *P*=0.025. (**g**) Hidden platform test (significances, see Supplementary Table 3) and (**h**) the probe test in the Morris water maze, *P*=0.003, 0.033. (**i**) Swim speeds of the probe test (crossing number of located hidden platform analysis, see Supplementary Fig. 2I). (**j**) Dose-dependent evaluation of EPPS-induced memory alterations. EPPS was orally given to 12-month-old APP/PS1 TG male mice in 0, 0.1, 1 or 10 mg kg^−1^ per day (*n*=7–9) dosages for 3 months. Per cent alternation on Y-maze (*P*=0.005, 0.041). The error bars represent the s.e.m. One-way analysis of variance followed by Bonferroni's *post-hoc* comparisons tests were performed in all statistical analyses (*P<0.05, **P<0.01, ***P<0.001; other comparisons were not significant).

To test the therapeutic efficacy of orally administered EPPS in a symptomatic transgenic (TG) animal model of AD, we used aged APPswe/PS1-dE9 (amyloid precursor protein/presenilin protein 1 (APP/PS1)) double-TG model mice (10.5-month-old, male; Fig. 2a). The APP/PS1 model produces elevated levels of human Aβ by expressing mutant human APP and PS1. This model is known to develop AD-like phenotypes from 5 months of age^15^. Before EPPS administration, we observed severe cognitive deficits and large amounts of plaques in the 10.5-month-old APP/PS1 mice (male, *n*=13) by Y-maze tests (*P*<0.0001; Fig. 2b) and brain plaque staining [Fig f3](Fig. 4a), respectively.

EPPS was included in drinking water, starting at 10.5 months until 14 months of age. After the daily oral administration of EPPS (10 and 30 mg kg^−1^ per day; *n*=11 and 8, respectively) or water (non-treated, *n*=15) for 3 months, mice were subjected to behavioural analyses, to monitor cognitive function. EPPS was continuously administered over the span of 2 weeks during the Y-maze, fear conditioning and Morris water maze tests (Fig. 2a). For comparison, age-matched WT mice (14-month-old, male, *n*=16) were also subjected to behavioural tests. These behavioural tests examine hippocampal functionality, which is one of the earliest and most acutely affected brain regions in AD^16^. Cognitive ability was determined as the per cent alternation on the Y-maze. We found that EPPS treatment significantly improved behavioural performance on the Y-maze test when compared with non-treated, age-matched TG mice (treatment effect, *P*=0.008 and 0.010; 10 and 30 mg kg^−1^ per day, respectively; Fig. 2c). The total number of individual arm entries was not altered by EPPS treatment; all cohorts of mice performed equally well in the Y-maze test (Fig. 2d). Next, the emotion-associated learning ability based on amygdala–hippocampal communication was assessed by measuring the freezing response in fear-conditioning tests. EPPS treatment was also found to significantly improve the performance of TG mice in the contextual fear-conditioning tasks compared with non-treated TG controls and was improved to a level similar to that of the WT mice (treatment effect, *P*=0.036 and 0.038; 10 and 30 mg kg^−1^ day, respectively; Fig. 2e). We did not observe significant behavioural improvement in APP/PS1 by EPPS in cued fear-conditioning tasks (Fig. 2f). Finally, in the Morris water maze task, EPPS-treated TG mice also showed significant cognitive recovery compared with non-treated, age-matched TG mice (treatment effects: 30 mg kg^−1^ day; 4-day training effect, *P*=0.088; 6-day training effect, *P*=0.019; Fig. 2g–i, Supplementary Fig. 2I and Supplementary Table 3), suggesting that EPPS treatment ameliorates spatial memory deficits in this model. As we did not observe dose-dependent therapeutic efficacy between EPPS-treated groups with 10 and 30 mg kg^−1^ day doses, we lowered the administration dosages of EPPS to 0, 0.1, 1 and 10 mg kg^−1^ day, and orally administered these doses to 12-month-old APP/PS1 mice for 3 months (male, *n*=8, 7, 8 and 9; 0, 0.1, 1 and 10 mg kg^−1^ day, respectively). In the Y-maze test, EPPS improved cognitive deficits in a dose-dependent manner in these mice (treatment effect, *P*=0.005 and 0.041; 0 versus 10 mg kg^−1^ day and 0.1 versus 10 mg kg^−1^ day, respectively; Fig. 2j).

**Figure 3 f3:**
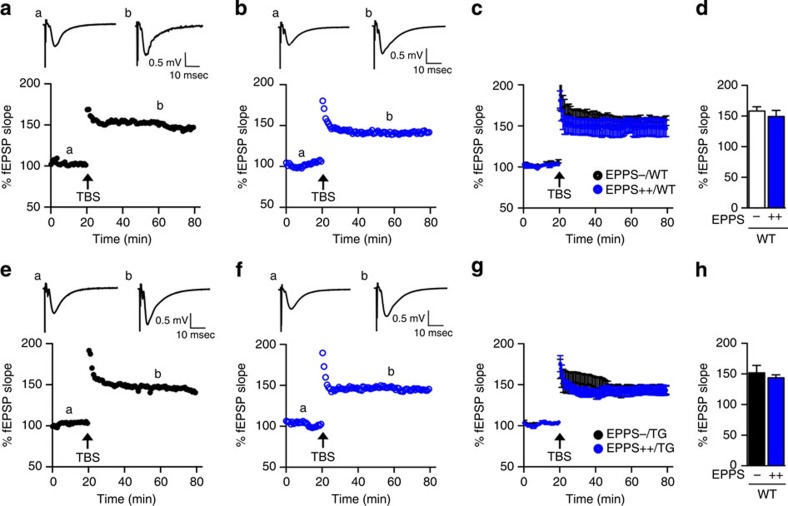
EPPS does not affect synaptic plasticity in mice. EPPS, 0 (EPPS**−**) or 30 mg kg^-1^ per day (EPPS**++**), was orally given to WT (*n*=4) and APP/PS1 TG (*n*=4) mice for 5 days. (**a**–**d**) LTP, measured by % field EPSP% fEPSP slope, in CA1 of hippocampal slices (three slices per mouse) from WT mice. (**a**) Upper trace, a representative trace of fEPSP before and after inducing LTP by pairing stimuli in CA1 of the hippocampus in non-treated mice. Lower trace, a time course of EPSP before and after inducing LTP by pairing stimuli in CA1 of the hippocampus in non-treated mice. (**b**) Upper trace, a representative trace of EPSP before and after inducing LTP by pairing stimuli in CA1 of the hippocampus (three slices per mouse) in EPPS-treated mice. (**c**) Averaged time course of EPSP before and after inducing LTP by pairing stimuli in CA1 of the hippocampus in non-treated and EPPS-treated mice. (**d**) Quantification of the effect of EPPS on LTP. (**e**–**h**) LTP, measured by the % fEPSP slope, in CA1 of hippocampal slices from TG mice. (**e**) Upper trace, a representative trace of EPSP before and after inducing LTP by pairing stimuli in CA1 of the hippocampus in non-treated mice. Lower trace, a time course of EPSP before and after inducing LTP by pairing stimuli in CA1 of the hippocampus in non-treated mice. (**f**) Upper trace, a representative trace of EPSP before and after inducing LTP by pairing stimuli in CA1 of the hippocampus in EPPS-treated mice. (**g**) Averaged time course of EPSP before and after inducing LTP by pairing stimuli in CA1 of the hippocampus in saline-treated and EPPS-treated mice. (**h**) Quantification of EPPS effect on LTP in TG mice. LTP was induced by theta burst stimulation (TBS), represented by the arrow. The error bars represent the s.e.m. Student's unpaired *t*-tests were performed in statistical analyses; comparisons were not significant (*P*>0.05).

To address the possibility that EPPS directly activates the cognitive abilities of mice without altering the APP/PS1-related neuropathology of AD, we examined changes in the cognitive behaviours and synaptic plasticity of WT C57BL/6 mice. When we orally administered EPPS to 10.5-month-old WT mice for 3.5 months (male, *n*=10 per group), we did not observe cognitive enhancement in Y-maze, fear-conditioning and Morris water maze tests (Supplementary Fig. 2A–H). To physiologically assess the effects of EPPS on long-term potentiation (LTP) in the WT (Fig. 3a–d) and APP/PS1 mice (Fig. 3e–h), we performed electrophysiology experiments and measured excitatory postsynaptic potential (% EPSP) in acute hippocampal slices (three slices per mouse) prepared from EPPS- and water-administered mice. At the Schaffer collateral (SC) inputs to hippocampal CA1, LTP of both WT (% EPSP increase by pairing stimuli: 158.2±7.1% for non-treated group (EPPS−/WT), *n*=4; 149.3±10.1% for treated group (EPPS++/WT), *n*=4) and APP/PS1 (% EPSP increase by pairing stimuli: 152.0±12.3% for non-treated group (EPPS−/TG), *n*=4; 143.6±5.0% for treated group (EPPS++/TG), *n*=4) mice was not affected by orally administered EPPS (30 mg kg^−1^ per day for 5 days) compared with non-treated controls (Fig. 3). These results imply that EPPS neither affects LTP nor enhances general learning and memory abilities of cognitively normal mice.

Collectively, these results indicate that EPPS rescues hippocampus-dependent cognitive deficits when orally administered to aged, symptomatic APP/PS1 TG mice.

### EPPS removes Aβ plaques and oligomers in APP/PS1 mice

**Figure 4:**
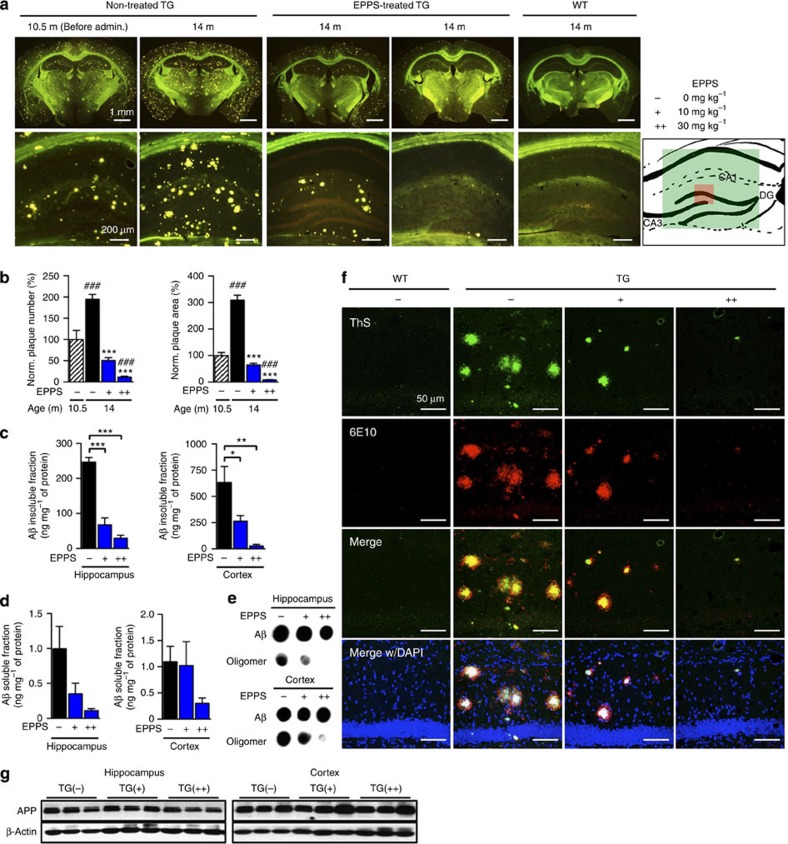
EPPS disaggregates Aβ plaques and oligomers in APP/PS1 mice. APP/PS1 mice and WTs from the aforementioned behavioural tests were killed and subjected to brain analyses. EPPS, 0 (TG(**−**), male, *n*=15), 10 (TG(**+**), male, *n*=11) or 30 mg kg^-1^ per day (TG(**++**), male, *n*=8), was orally given to 10.5-month-old APP/PS1 for 3.5 months and their brains were compared with age-matched WT brains (WT(**−**), male, *n*=16). (**a**) ThS-stained Aβ plaques in whole brains (scale bars, 1 mm) and the hippocampal region (scale bars, 200 μm) of each group. The mouse brain schematic diagram was created by authors (green and red boxes: regions of brain images, **a** and **f**, respectively). (**b**) Number or area of plaques normalized (%) to the level in 10.5-month-old TG mice. Plaque number: *P-*values compared with TG (male, 10.5-month-old) are all <0.0001 (#). *P-*values compared with TG(**−**) (male, 14-month-old) are all <0.0001 (*). Plaque area: *P-*values compared with TG (male, 10.5-month-old) are all <0.0001 (#). *P-*values compared with TG(**−**) (male, 14-month-old) are all <0.0001 (*). (**c**–**e**) Aβ-insoluble and -soluble fractions analyses from brain lysates. (**c**) Sandwich ELISA of Aβ-insoluble fractions. Hippocampus: all *P*<0.0001; cortex: *P*=0.004, 0.046. (**d**) Sandwich ELISA of Aβ-soluble fractions. (**e**) Dot blotting of the total Aβ (anti-Aβ: 6E10, also recognizes APP) and oligomers (anti-amyloidogenic protein oligomer: A11). (**f**) Histochemical analyses of Aβ deposition. Aβs were stained with the 6E10 antibody and ThS. Aβ plaques (first row): green; all Aβs (second row): red; 4,6-diamidino-2-phenylindole (DAPI): blue (as a location indicator). The third and bottom rows show merged images of plaques and Aβs, and plaques and Aβs with DAPI staining. Scale bars, 50 μm. (**g**) Western blotting analyses of APP expression in hippocampal and cortical lysates (detected at ∼100 kDa by 6E10 antibody). Densitometry (see Supplementary Fig. 3A). Full version (see Supplementary Fig. 7). The error bars represent the s.e.m. One-way analysis of variance followed by Bonferroni's *post-hoc* comparisons tests were performed in all statistical analyses (*P<0.05, **P<0.01, ***P<0.001, ^#^*P*<0.05, ^##^*P*<0.01, ^###^*P*<0.001; other comparisons were not significant).

All of the aforementioned APP/PS1 and WT mice were killed after behavioural tests and had their brains examined in histochemical and blotting analyses. To examine the effect of orally administered EPPS on the amount of Aβ plaques and oligomers in the brain, brains of non-treated APP/PS1 mice were sectioned and then stained with thioflavin S (ThS) to visualize dense-core Aβ plaques^17^ (Fig. 4a). Compared with 10.5-month-old APP/PS1 brains, we observed a significant increase in Aβ plaques, twofold in number and threefold in area, throughout the entire brain of 14-month-old APP/PS1 mice (*P*<0.0001; Fig. 4a,b). By contrast, EPPS treatment reduced the levels of Aβ plaques in APP/PS1 mice in a dose-dependent manner (treatment effect, *P*<0.0001 for both 10 and 30 mg kg^−1^ per day; Fig. 4a,b) and substantially eliminated Aβ plaques in the hippocampus at the dose of 30 mg kg^−1^ per day (EC_50_=5.22 mg kg^−1^ per day; Fig. 4b).

As an independent measure of Aβ levels, we performed sandwich enzyme-linked immunosorbent assay (ELISA; Fig. 4c,d). To separately analyse levels of insoluble and soluble Aβ, hippocampal and cortical regions were lysed using guanidine (insoluble fraction) or sucrose lysis (soluble fraction) buffers. As a result, we found a dose-dependent reduction of insoluble Aβ in the brains of EPPS-treated APP/PS1 mice (hippocampus, *P*<0.0001 for both 10 and 30 mg kg^−1^ per day; cortex, *P*=0.046 and 0.004, 10 and 30 mg kg^−1^ per day, respectively; Fig. 4c), which was similar to ThS histochemistry results (Fig. 4a,b). On the contrary, we did not observe a significant decrease in the level of soluble Aβ species by EPPS (Fig. 4d). Soluble Aβ in the sucrose lysis buffer could comprise both monomers and oligomers. To assess the level of Aβ oligomers, we performed dot blot assays on brain lysates using 6E10 and A11 antibodies. The 6E10 antibody detects all Aβ species and recognizes APP, whereas A11 detects oligomeric proteins in soluble fractions. EPPS dose dependently decreased the amount of oligomeric species but the concentration of total soluble Aβ, which includes monomers, did not change (Fig. 4e).

Previous clinical investigations suggested that ThS-negative diffuse Aβ plaques were an early pathological sign of AD^18^,^19^. To eliminate the possibility that EPPS only loosened β-sheet-rich aggregates into less dense diffuse plaques, we performed Aβ immunohistochemistry using the anti-Aβ 6E10 antibody^20^. In contrast to ThS, 6E10 can stain both dense core and diffuse plaques in brain tissues. Fluorescent microscopy illustrated that ThS- and 6E10-stained plaques precisely co-localized in the hippocampal region and were equally reduced by EPPS treatment in a dose-dependent manner (Fig. 4f). We did not observe ThS-negative diffuse plaques in EPPS-treated APP/PS1 mice. To examine whether the oral administration of EPPS affected the expression levels of APP, we performed western blot analyses. In the hippocampal and cortical regions of APP/PS1 mice, the levels of APP were similar between the water- and EPPS-treated groups (Fig. 4g and Supplementary Fig. 3A). Neuropathological development of AD based on gender differences has previously been reported^21^,^22^. Female APP/PS1 mice deposit Aβ earlier than age-matched male mice and the cause of the difference is unclear. To examine whether EPPS exerted similar effects on female mice, EPPS (30 mg kg^−1^ per day) was included in drinking water and provided to female APP/PS1 mice (10.6-month-old, *n*=6 per group) for 1 month. Consistent with the results from male APP/PS1 mice, EPPS treatment similarly and significantly reduced Aβ plaques in the brains of female TG mice (plaque number and area, *P*<0.0001; Supplementary Fig. 4). Collectively, these results indicate that orally administered EPPS effectively decreases Aβ plaques and oligomers in APP/PS1 model mouse brains.

### EPPS lowers Aβ-dependent inflammation and glial GABA release

**Figure 5:**
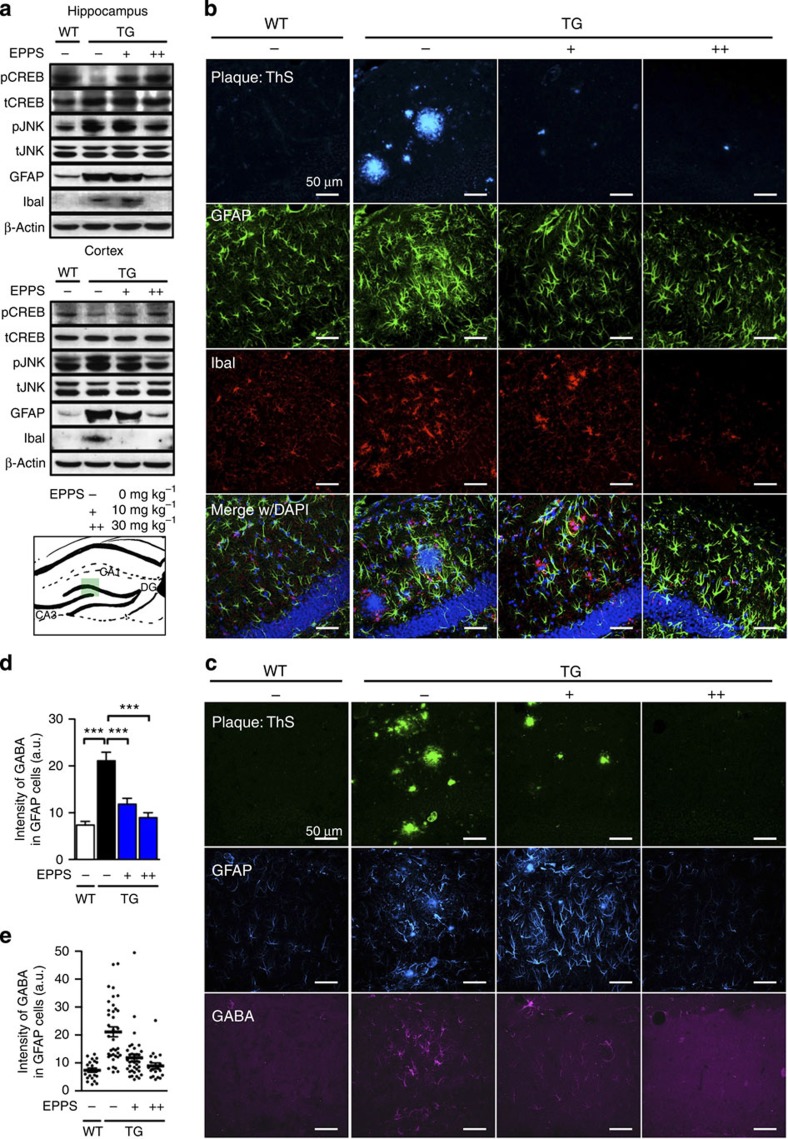
EPPS lowers inflammation and glial GABA release. APP/PS1 and WT mice from the aforementioned behavioural tests were killed and subjected to brain analyses. EPPS, 0 (TG(**−**), male, *n*=15), 10 (TG(**+**), male, *n*=11) or 30 mg kg^−1^ per day (TG(**++**), male, *n*=8), was orally given to 10.5-month-old APP/PS1 for 3.5 months and their brains were compared with age-matched WT brains (WT(**−**), male, *n*=16). (**a**) Western blotting of phosphorylation of cyclic AMP response element-binding protein (pCREB), pJNK, GFAP and Iba-1 (densitometry, see Supplementary Fig. 3B, C). Full version (see Supplementary Fig. 8). (**b**–**e**) Histochemical analyses of Aβ deposition, GFAP, Iba-1 and GABA. (**b**) Aβ plaques stained with ThS (first row): blue; GFAP (second row): green; Iba-1 (third row): red; and 4,6-diamidino-2-phenylindole (DAPI; fourth row): blue (as a location indicator). The bottom row shows merged images of plaques, GFAP and Iba-1 with DAPI staining. (**c**) Aβ plaques stained with ThS (first row): green; GFAP (second row): blue; and GABA (third row): violet. Scale bars, 50 μm. (**d**) Quantification of GABA in confocal images^25^. Each dot represents the number of GFAP-positive cells with GABA; a.u., arbitrary unit. (**e**) GABA average from the previous panel (*P*<0.0001 for all). Values refer to GFAP-positive GABA. The error bars represent the s.e.m. One-way analysis of variance followed by Bonferroni's *post-hoc* comparison tests were performed in the statistical analysis (***P<0.001; other comparisons were not significant). The mouse brain schematic diagram was created by authors (green box: region of brain images).

Aβ-dependent inflammation generally reflects the extent of injury and toxicity in AD^23^. Thus, we examined whether EPPS treatment affected inflammation by immunohistochemistry of male APP/PS1 mouse brains from the aforementioned behavioural studies. EPPS treatment markedly reduced the levels of c-Jun N-terminal kinase (JNK) phosphorylation, astrocytosis (GFAP, glial fibrillary acidic protein) and microgliosis (Iba-1, ionized calcium-binding adaptor molecule-1) to the level of WT mice. On the contrary, the phosphorylation of cyclic AMP response element-binding protein, which is related to memory enhancement in AD, was increased^2^,^24^ (Fig. 5a and Supplementary Fig. 3B,C). In addition, large reductions in plaques, astrocytosis and microgliosis were confirmed by immunohistochemistry (Fig. 5b). These data show that the aberrant elevation of inflammation found in APP/PS1 TG mice was reduced after oral administration of EPPS.

Previously, we reported that reactive astrocytes abundantly produced and released the inhibitory gliotransmitter γ-aminobutyric acid (GABA) by the stimulation of Aβ aggregates, and that the suppression of tonic glial GABA release recovered synaptic plasticity and cognitive deficits in APP/PS1 TG mice^25^. In this study, we measured EPPS-induced alterations of the tonic GABA release from reactive astrocytes in aged APP/PS1 mice by immunohistochemistry (Fig. 5c). We observed that daily treatment of EPPS (30 mg kg^−1^ per day) significantly reduced the levels of GABA around reactive astrocytes (GFAP stained) in the hippocampal region of APP/PS1 mice to a level similar to that of WT (treatment effects, *P*<0.0001 for both 10 and 30 mg kg^−1^ per day, Fig. 5d,e). These results support the view that the elimination of Aβ plaques by agents such as EPPS suppresses the abnormal tonic release of GABA from reactive astrocytes and recovers memory impairments.

### EPPS disaggregates Aβ oligomers and fibrils by direct interaction and reduces cytotoxicity

**Figure 6:**
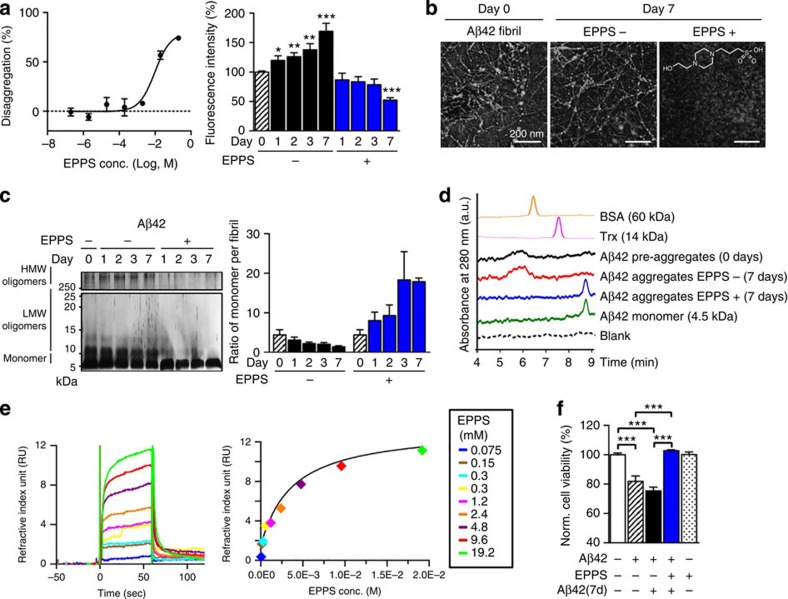
EPPS disaggregates Aβ aggregates by selective binding. (**a**–**c**) Preformed Aβ42 aggregates (25 μM, Day 0) were incubated with EPPS. (**a**) EPPS concentration-dependent (200, 20, 2, 0.2, 0.02, 0.002, 0.0002 or 0 mM of EPPS, 3-day treatment) and incubation time-dependent (20 mM EPPS for 1, 2, 3 and 7 days) ThT assays. Fluorescence intensity was normalized to preformed Aβ aggregates (100%, Day 0). Statistical comparisons were made with day 0 (*n*=4, Student's *t*-test; from the left: *P*=0.034, 0.004, 0.005, 0.000, 0.000). (**b**) Transmission electron microscopic images of EPPS-induced Aβ fibril disassembly. Scale bars, 200 nm. The inset shows the chemical structure of EPPS. (**c**) Silver staining for the SDS–PAGE analysis of PICUP cross-linked Aβ aggregates and the densitometry analysis in the ratio of monomer to fibril (HMW, high molecular weight; LMW, low molecular weight). Full-length version (see Supplementary Fig. 9). (**d**) SEC analysis. Size markers: BSA (yellow) and thioredoxin (Trx, pink). Control: Aβ42 monomer (green). a.u., arbitrary unit. (**e**) Surface plasmon resonance analyses. Dose-dependent kinetics of EPPS targeting Aβ40 oligomers and the corresponding fitting curve from the saturated region of the sensorgram. (**f**) MTT assays. Aβ42: 2.5 μM Aβ42 aggregates, Aβ42(7d): 2.5 μM Aβ42 aggregates were incubated for 7 days with/without EPPS (2 mM). HT-22 cells were treated with the prepared samples for 24 hours. Cell viability was normalized to that of the non-treated cells (100%). All *P*-values were <0.0001 (*n*=5). The error bars represent the s.e.m. of independent triplicate measurements. One-way analysis of variance followed by Bonferroni's *post-hoc* comparison tests were performed in the statistical analyses (*P<0.05, **P<0.01, ***P<0.001; other comparisons were not significant).

To understand how EPPS reduces amyloid deposits and recovers cognitive deficits in mouse models, we investigated interactions between EPPS and Aβ aggregates by *in vitro* biochemical and biophysical assays. Previously, we reported that EPPS inhibited the *de novo* formation of Aβ oligomers and fibrils in thioflavin-T (ThT), SDS–polyacrylamide gel electrophoresis (SDS–PAGE) and transmission electron microscopy^13^. In this study, we prepared Aβ oligomers and fibrils through the preincubation of the peptide and monitored EPPS-induced alterations to these aggregates using ThT, SDS–PAGE and transmission electron microscopy. We performed a cell-free ThT fluorescence assay to detect ThT bound to a β-sheet complex, which is proportional to the amount of Aβ fibril^10^,^26^. Preformed aggregates of the two most common Aβ types, Aβ42 and Aβ40, were incubated with or without candidate molecules for 1, 2, 3 and 7 days. EPPS dose dependently disaggregated β-sheet-rich preformed Aβ fibrils (Fig. 6a and Supplementary Fig. 5A). The ThT fluorescence assay can produce false-positive results when, for example, EPPS binds to ThT and interferes with the complex formation between ThT and Aβ fibrils, leading to a decrease in ThT fluorescence intensity^26^. To circumvent this issue, we directly visualized insoluble Aβ fibrils using transmission electron microscopy in the presence and the absence of EPPS. We found that a 7-day treatment of EPPS completely disaggregated the hair-like Aβ fibril structures (Fig. 6b and Supplementary Fig. 5B). Among Aβ aggregates, soluble oligomers, including dimers and trimers, are reported to be the most neurotoxic species^3^,^27^,^28^. To test whether EPPS disaggregates harmful Aβ oligomers into non-toxic monomers, we performed SDS–PAGE with photo-induced cross-linking of the unmodified proteins (PICUP), followed by silver staining, which allows us to separate and compare the assembled oligomeric species^29^. We found that EPPS treatment sharply reduced high-molecular-weight aggregates (above 250 kDa) and oligomeric species, while increasing the concentration of monomers, suggesting that EPPS may disaggregate Aβ42 and Aβ40 aggregates (Fig. 6c, Supplementary Fig. 1B and Supplementary Fig. 5C). As SDS provides a denaturing environment that may allow for the dissociation of protein aggregates on a gel in spite of cross-linking, we used size-exclusion chromatography (SEC) on high-performance liquid chromatography, to confirm oligomer dissociation by EPPS. Using samples identical to ones subjected to the aforementioned PICUP–SDS experiments, we analysed the changes in Aβ42 oligomer concentrations. Preformed aggregates, aggregates that were incubated for 7 days (EPPS−) and aggregates that were incubated for 7 day with EPPS treatment (EPPS+) were serially injected into the SEC column, with BSA (60 kDa), thioredoxin (14 kDa) and Aβ42 monomers (4.5 kDa) as reference size markers (Fig. 6d). All samples were not cross-linked and insoluble species were excluded by filtering before injection. We observed that Aβ42 oligomers larger than 60 kDa were the major component of both preformed aggregation and aggregates with the 7-day additional incubation. Consistent with the results from the electrophoresis study, EPPS treatment lowered the level of these oligomers and increased the amount of monomers.

The reduced amount of oligomers and fibrils strongly suggested that EPPS directly binds to Aβ aggregates. To assess direct interaction between EPPS and Aβ aggregates, we employed a label-free surface plasmon resonance test to determine the bimolecular binding kinetics^30^. We immobilized Aβ40 aggregates and monomers on a CM5 sensor chip surface via amine coupling and allowed EPPS in various concentrations (from 0.075 to 19.2 mM) to pass over the Aβ-coupled surface in a Biacore T200 system. Formation of Aβ40 aggregates was confirmed by SDS–PAGE with PICUP cross-linking before the surface immobilization. Reduction in the intensity of reflected light from the chip on binding and dissociation between EPPS and Aβ was measured. We found that EPPS directly bound to Aβ aggregates, as evidenced by the rapid increase of the binding response during EPPS injection in a dose-dependent manner (Fig. 6e). Although the heterogeneous nature of Aβ aggregates made the calculation of the precise binding constant unfeasible, the substantial curve fitting efficiency (0.286 RU^2^ of calculated *χ*^2^) supported the dose-dependent interaction between EPPS and immobilized Aβ aggregates^31^. On the contrary, no significant interaction between EPPS and Aβ monomers was observed (Supplementary Fig. 5D).

To examine the toxicity of EPPS-treated Aβ aggregates, we performed cell viability assays on a cultured mouse hippocampal cell line (HT-22). We prepared preformed Aβ aggregates (Aβ42=2.5 μM and Aβ40=5 μM) and then treated the HT-22 cells with these aggregates for 24 hours. In addition, the preformed Aβ aggregates were incubated with or without EPPS for 7 days and then the cells were treated with these pretreated aggregates for 24 hours (final concentration of Aβ42(7d)=2.5 μM, Aβ40(7d)=5 μM, EPPS=2 mM). EPPS treatment fully rescued the cells from death induced by Aβ42 (Fig. 6f) and Aβ40 (Supplementary Fig. 5E). EPPS alone did not induce any significant cell damage (Fig. 6f and Supplementary Fig. 5E). As oxidative stress is associated with cellular and synaptic dysfunctions in AD brains, we performed a 1,1-diphenyl-2-picryl-hydrazyl assay^32^ to assess whether EPPS acts as an antioxidant. However, we did not observe any free radical scavenging properties of EPPS in the assay (Supplementary Fig. 6). Taken together, these results suggest that EPPS markedly disaggregates both toxic oligomers and fibrils into monomers and prevents Aβ-induced cell damage by direct binding to Aβ aggregates.

## Discussion

Here we report that (1) a small molecule, EPPS, converts neurotoxic oligomers and plaques into non-toxic monomers by directly binding to Aβ aggregates; (2) orally administered EPPS produces a dose-dependent reduction of Aβ plaque deposits and behavioural deficits in APP/PS1 TG mice, even when administration was delayed until after the pathology was well established; (3) the beneficial effect of EPPS probably operates through an Aβ-related mechanism rather by facilitating cognitive processes; and (4) large doses of EPPS appeared to be well tolerated in initial toxicity studies^6^,^7^,^33^. EPPS may integrate the therapeutic advantages of previously described Aβ-clearing antibodies with the advantages of anti-amyloidogenic chemicals as a small molecule capable of disaggregating diverse species of oligomeric and fibrillar Aβ, ameliorating the development of Aβ plaque pathology and behavioural deficits in APP/PS1 TG mice with early treatment and rescuing the pathology and behavioural deficits with delayed administration. Among the three mechanisms of anti-Aβ immunotherapeutics (solubilization by direct binding to Aβ, phagocytosis by activated microglia and activation of Aβ extraction to the blood by peripheral sink), the therapeutic behaviour of EPPS most resembles the solubilization by direct binding to Aβ. This strategy recently showed promising clinical indications for preventing or treating AD^34^,^35^. Furthermore, the disaggregating mode of action of EPPS generates and leaves monomers that may be essential for physiological brain function^8^,^36^,^37^,^38^,^39^. A recent study reported benefits of Aβ peptides in a multiple sclerosis mouse model by modulating the autoimmune neuroinflammation^40^. Blood-brain barrier permeability and lower manufacturing cost are also considerable benefits of EPPS compared with antibody drug candidates. Additional studies are warranted to determine whether these favourable actions of EPPS and derivatives will translate into a therapy that might potentially be useful across a range of AD stages.

The present data further suggest that the disaggregating mode of action of EPPS should be explored in detail. First, the effect of EPPS treatment on cognitive behaviours and neuropathological biomarkers in APP/PS1 mice showed different responses based on dosage. Although the levels of Aβ oligomers and plaques in the brain were reduced in a dose-dependent manner within 10, 30 and 100 mg kg^−1^ per day treated groups, we did not observe significant differences between these groups in the behavioural studies. Structurally, EPPS shares its amino sulfonic acid group with well-known Aβ-regulating molecules, such as tramiprosate and bis-ANS^5^,^13^,^41^,^42^. The sulfonic acid group has been reported to mimic the properties of the sulfate ion that is required for the strong binding of glycosaminoglycans to the HHQK region of Aβ^5^,^41^. In our previous study, we also found that HEPES containing 2-(4-ethylpiperazin-1-yl)ethanol, which is one carbon short of 2-(4-propylpiperazin-1-yl)ethanol, directly inhibits the formation of Aβ aggregates^13^. However, a further in-depth structure–activity relationship study is required to understand how EPPS selectively targets and disaggregates Aβ oligomers and plaques. It is notable that EPPS requires a relatively higher dose *in vitro* than *in vivo*, to disaggregate preformed oligomers and plaques. One explanation of this finding is the requirements for highly concentrated Aβ to be detected in *in-vitro* conditions^5^,^43^,^44^. Compared with the nanomolar concentration of Aβ in the AD brain, a micromolar or even higher concentration of the peptide is needed to visualize aggregates. Thus, the dose of EPPS also increases to disaggregate these aggregates. Another possible hypothetic explanation is the lack of Aβ clearance mechanisms in *in-vitro* experiments. In the brain, detached Aβ monomers from aggregates by EPPS may simultaneously become subjected to multiple clearance pathways including the peripheral sink and microglial activation^45^. We suspect that a higher population of EPPS is required *in vitro*, to disaggregate pre-existing and newly forming Aβ aggregates. In addition, considering that EPPS is one of the commonly used buffer substances for biological experiments, we highly recommend not using EPPS buffers for amyloid studies.

Over the last three decades, there has been an ongoing controversy of whether Aβ accumulation is a cause or rather an effect of AD^8^. In this study, we demonstrated that AD-related learning and memory deficits in a TG mouse model of AD are ameliorated by an agent capable of disaggregating Aβ oligomers and fibrils. Therefore, our study supports the view that Aβ aggregation is a direct driver of AD symptoms.

## Methods

### Animal studies

*Ethical regulations*. All animal experiments were carried out in accordance with the National Institutes of Health guide for the care and use of laboratory animals (NIH Publications No. 8023, revised 1978) and the Animal Institutional Animal Care and Use Committee of KIST (Seoul, Korea).

*Preparation of memory-impaired mice by Aβ aggregates and treatment of EPPS*. Briefly, male ICR mice (8-week-old) were purchased from Orient Bio Inc. (Seoul, Korea) and habituated for 4 days. Aβ42 (10 μM) in PBS (10% dimethyl sulfoxide (DMSO)) was incubated at 37 °C for 1 week, to obtain various soluble oligomeric species, and then 5 μl of Aβ or vehicle (90% PBS and 10% DMSO) were acutely injected into the intracerebroventricle^47^. Aβ42 for injection was purchased from American Peptide Company. Experiments were designed in two paradigms (*n*=9–10 per group): oral administration of EPPS (30 or 100 mg kg^−1^ per day) (1) before (pretreatment) or (2) after Aβ injection (co-treatment) into mice. (1) EPPS was administered for 1 week before the Aβ injection into the intracerebroventricle. EPPS was administered for another week following injection and the behavioural test (Y-maze) was performed. (2) Aβ was injected on the same day when EPPS administration began for following 5 days and the behavioural test (Y-maze) was performed.

*Toxicity*. EPPS was orally administered to B6 mice (4-week-old, male, *n*=6 per group) as 1,000 or 2,000 mg kg^−1^ per day for 2 months, to obtain the LD_50_ of EPPS. During administration of EPPS, changes in mortality, body weight and hair loss of mice were observed.

*Pharmacokinetics of EPPS*. To evaluate pharmacokinetic profiles, EPPS was administered to SD rats via drinking water for 7 consecutive days (day 1–7) for test groups (Medicilon Preclinical Research, Shanghai LLC, Study number: 11006–14013). We measured plasma and brain concentrations of EPPS at different time points (24 (only for plasma), 70, 120 and 168 hours) from individual animals following oral administration (10 and 100 mg kg^−1^ per day). Eighteen SD male rats (body weight: 185.6–198.1 g) were placed under observation. The amount of drinking water that the study animals had drunk for a day (∼24 hours) was checked and averaged at around 1,000 hours each day. Following administration of EPPS at a dose of 10 mg kg^−1^ per day, the concentrations of plasma EPPS were 2.44±0.32, 7.00±2.42, 3.70±0.66 and 4.30±1.91 ng ml^−1^ at each time point (24, 70, 120 and 168 hours). Brain EPPS concentrations were 7.52±2.85, 7.03±1.83 and 7.52±3.40 ng ml^−1^ at each time point (70, 120 and 168 hours). When EPPS was administered at a dose of 100 mg kg^−1^ per day, the concentrations of plasma EPPS were 23.73±12.21, 79.48±22.15, 63.12±23.65 and 33.74±5.39 ng ml^−1^ at each time point (24, 70, 120 and 168 hours) and brain EPPS were 17.86±11.88, 9.15±2.83 and 11.32±1.03 at each time point (70, 120 and 168 hours).

*Preparation of APP/PS1 double TG mice*. Double mutated TG mice were originally obtained from Jackson Laboratory (USA; strain name: B6C3-Tg (APPswe, PSEN1dE9) 85Dbo/J; stock number 004462). These APP/PS1 TG mice were maintained as double hemizygotes by crossing with WT mice on a B6C3F1 background strain.

*Oral administration of EPPS to APP/PS1 double TG mice*. EPPS in drinking water was freely administered to 10.5-month-old WT (male) and APP/PS1 TG (male) mice, with severe cognitive deficits and plaque deposits in brains, for 3.5 months with 10 or 30 mg kg^−1^ per day dosages (*n*=8–11 per group). For control groups, water-treated TG mice (male, *n*=15) and age-matched WTs (male, *n*=16) were used. For dose–response studies, we administered EPPS (0, 0.1, 1 or 10 mg kg^−1^ per day) to 12-month-old APP/PS1 TG mice (male) for 3 months; 0 (*n*=8), 0.1 (*n*=7), 1 (*n*=8) or 10 mg kg^−1^ (*n*=9). To determine the exact dosage, all administered molecules in drinking water were set according to the average daily consumption in each cage and recalculated once a week. Animal tests were employed twice independently and all behavioural tests were performed during the light cycle.

*Electrophysiology*. To obtain LTP with or without EPPS administration, we prepared APP/PS1 TG (*n*=4) and WT (*n*=4) mice, and administered water (EPPS−, *n*=2 per group) or 30 mg kg^−1^ per day of EPPS (EPPS++, *n*=2 per group) for 5 days in drinking water then compared LTP (BnH, Gyeonggi, Korea). To investigate CA1 circuit from transverse SC input in hippocampal slices (400 μm thick), three slices of brain section per each mouse were prepared from adult mice. Briefly, after mice were anaesthetized with isoflurane, the brain was rapidly cooled via transcardiac perfusion with ice-cold sucrose-artificial cerebrospinal fluid (CSF). The brain was removed and placed in ice-cold sucrose-artificial CSF. Coronal slices were prepared to be incubated in artificial CSF at 35 °C for 30 min to recover. Slices were then incubated in artificial CSF at room temperature (23–25 °C) for 1–4 hours before being placed in the recording chamber for experiments. The standard artificial CSF contained (in mM) 119 NaCl, 2.5 KCl, 2.5 CaCl_2_, 1.3 MgSO_4_, 1.0 NaH_2_PO_4_, 26.2 NaH_2_CO_3_, 11 glucose, 1 Na pyruvate, 0.4 Na ascorbate saturated with 95% O_2_ and 5% CO_2_. Sucrose-artificial CSF contained (in mM) 198 sucrose, 2.5 KCl, 1 NaH_2_PO_4_, 26.2 NaHCO_3_, 11 glucose, 1 Na pyruvate, 0.4 Na ascorbate saturated with 95% O_2_ and 5% CO_2_. All experiments were conducted at 27–29 °C. For electrophysiological experiments, electrodes with 3–6 MΩ pipette resistance were used and extracellular field recordings were obtained from the neurons under visual guidance using infrared–differential interference contrast optics guidance. CA3 and DG regions were cut away just before starting LTP experiments, to isolate CA1 lesion. Stimuli were applied to the SC pathway using a concentric bipolar electrode located 100–200 μm from the recorded cell soma. Recordings were made using a multiclamp 700A (Molecular Devices, Sunnyvale, CA), digitized at 10 KHz and filtered at 2 KHz. Input resistance and series resistance were monitored continuously during recordings. In LTP experiments, baseline EPSPs before applying pairing stimuli (2 Hz, 2 min stimuli and postsynaptic depolarization to 0 mV) were measured for 3 min. After pairing stimuli (2 Hz, 2 min stimuli and postsynaptic depolarization to 0 mV), EPSPs were collected every 10 sec for 30 min.

### Behavioural studies

*Recording and analyses*. Data analyses, including recordings of all behavioural responses, were transcribed manually into the computer-acceptable format by keeping research colleagues blind.

*Y-maze tests*. Y-maze tests are used to assess cognitive changes, short-term spatial working memory (by spontaneous alternation) and exploratory activity (by total number of arm choices) of mice placed into a black Y-maze^16^,^48^,^49^. The Y-maze is a three-arm horizontal maze (40 cm long and 10 cm wide with 12-cm-high walls) in which the arms are symmetrically disposed at 120° angles from each other. The task was carried out on day 1 of behavioural tests. Mice were placed at the end of one arm and allowed to move freely through the maze during an 8-min session. The number of total arm choices and sequence of arm choices were recorded. The per cent alternation is defined by proportion of arm choices that differ from the last two choices. Before each trial, the interior of the maze was sprayed with a 70% ethanol solution to erase any scent cues.

*Fear-conditioning tests*. Contextual and cued fear-conditioning tests measure the ability of mice to learn and remember associations between aversive experiences and environmental cues^16^,^50^,^51^. Hippocampal lesions interfere with contextual conditioning but not the cued, whereas amygdala lesions interfere with the conditioning of both, the context and the cue^52^,^53^. Thus, to obtain the functional recovery of the hippocampal and the amygdala lesions, both tasks (contextual and cued tasks) were carried out during days 4–7 of behavioural tests. The test was employed according to the previous methods^16^,^50^,^51^,^52^,^53^,^54^. On the first day of fear-conditioning tests, mice were placed into a cued box for 5 min to habituate and explore. The next day, for training (fear conditioning phase), mice were placed into a sound-attenuating standard operant chamber (Coulbourn, USA) for 3 min. A 30-sec tone (3 kHz, 85 dB) was delivered followed by 1 sec foot shock (0.5 mA). This training was repeated twice continuously within a 90-sec interval. After 24 hours, for contextual retention tests, mice were subjected to the same chamber of conditioning and freezing response was measured for 5 min. Responses were recorded on a video camera to score freezing, which is the lack of movement, except for respiration. Cued fear-conditioning tests were performed on the last day of the fear-conditioning study. Mice were placed into the cued box allowing exploration for 3 min and the conditioning tone was delivered for 1 min with measurement of freezing responses.

*Morris water maze tests*. The Morris water maze tests are used to evaluate spatial reference learning and memory of mice^16^,^55^,^56^. In this study, a circular water tank (120 cm diameter, 25±1 °C) with hidden quadrants was used and the surrounding was decorated with eye-catching visual cues to orient subjected mice. A clear 12 cm (diameter) escape platform was placed 1.5 cm below the water surface, which was made opaque with white non-toxic tempera paint. The quadrant containing the platform refers to the target zone. Hidden platform test was performed for 6 days. Mice were trained for 6 consecutive days with five trials per day as acquisition trials. Each trial began with placing the mouse into a different quadrant and allowing it to swim freely for a maximum period (60 sec). After each mouse reached the platform (or were guided to the platform if mice were unable to locate the platform after 60 sec), they were returned to their home cage to dry for 20 min. The time taken to reach the platform of each mouse refers to escape latency. On the seventh day, probe test was performed to determine memory retention without the platform for 60 sec. Each mouse was placed into the opposite quadrant of the target zone where the platform was removed (time in the target zone). Numbers of annulus crossing in probe were also calculated. Data were recorded and analysed using a video camera-based Ethovision System (Nodulus, The Netherlands).

### Biochemical studies

*ThS staining and histochemical analyses of Aβ plaques in brain sections*. After the behavioural studies, mice were deeply anaesthetized with 2% avertin (20 μg g^−1^, intraperitoneally) and perfused with 0.9% saline followed by ice-cold 4% paraformaldehyde. Excised brains were post-fixed overnight in 4% paraformaldehyde at 4 °C and immersed in 30% sucrose for 48 hours for cryoprotection. Coronal hippocampal sections (35 μm; average range: bregma −1.70 to −2.01 mm) were cut (eight slices of each mouse brain) with a Cryostat (Microm HM 525, Thermo Scientific, Waltham, MA, USA) and mounted onto glass slides. Aβ plaques in brains were visualized using ThS staining. ThS was dissolved in 50% of ethanol at 500 μM and brain sections were stained for 7 min. As a differentiation step to remove a nonspecific binding of the dye, a slide was soaked into 100, 95 and 90% ethanol solutions for 10 sec each and then moved into PBS. Aβ plaques, GABA and inflammation-related proteins such as GFAP or Iba-1 were stained with primary antibodies with fluorescence-labelling secondary antibodies. Detailed information about the antibodies is included in the Supplementary Information. The images of plaques were taken on an Olympus fluorescent microscope using fluorescent filter sets^57^. Numbers and area of plaques were obtained by Image-J programme (NIH). Analyses of plaque distributions were transcribed manually into the computer-acceptable format by keeping research colleagues blind.

*Blotting analyses and quantification of Aβ-soluble fraction of brain lysates*. Mice were killed and hippocampal and cortical regions of mouse brains were dissected separately. Brain tissues were homogenized in ice-cold lysis buffer (10 mM Tris-HCl, 5 mM EDTA in 320 mM sucrose pH 7.4) containing 1 × proteinase inhibitor cocktail^58^. Homogenized tissue was incubated in ice for 20 min and centrifuged at 14,000 r.p.m., 4 °C for 30 min. The supernatant (soluble fraction) of brain lysates was used in Aβ42 ELISA, western blots and dot blots for biochemical changes and soluble Aβ analyses, respectively. Concentrations of soluble fractions of brain lysates were determined by Bradford protein assay. Protein samples (20 μg) were loaded on each lane of SDS–PAGE gels for western blot analyses and used for Aβ42 sandwich ELISA (Aβ42 sandwich ELISA kit from Invitrogen). Measurements of Aβ42 in soluble fractions were performed according to the manufacturer's instructions, with fivefold diluted soluble fraction samples. Protein samples in soluble fraction of brain lysates, 20 and 1 μg, were spotted for the dot blot analysis to detect oligomers and Aβ, respectively. Total-CREB, p-CREB, Iba-1, total-JNK, p-JNK, GFAP, APP, Aβ (6E10), protein oligomers (A11) and β-actin (a loading control) were measured. The detail information of antibodies is included in Supplementary Information.

*Quantification of Aβ-insoluble fraction of brain lysates*. To obtain Aβ-insoluble fraction in brain lysates, a guanidine buffer (5 mM guanidine-HCl, 50 mM Tris-HCl pH 8.0) containing 1 × proteinase inhibitor cocktail was added to the pellet of brain lysates and the mixtures were incubated at room temperature for 3 hours with shaking to dissolve Aβ-insoluble fraction. After centrifugation at 4 °C for 2 hours, levels of dissolved Aβ in supernatant (insoluble fraction) were measured by Aβ42 sandwich ELISA kit purchased from Invitrogen (KHB3442). Protein concentrations of insoluble fractions were determined by Bradford protein assay and 10 μg of protein samples were used. Measurements were performed according to the manufacturer's instructions.

### *In vitro* studies

*Preparation of Aβ aggregates*. In-house synthetic Aβ peptides were dissolved in DMSO to prepare Aβ40 (50 mM) and Aβ42 (25 mM) stocks^13^,^59^. Each stock was then diluted with deionized water to make Aβ solutions, Aβ40 (500 μM) and Aβ42 (250 μM). Aβ solutions were incubated for 5–10 days at 37 °C to obtain Aβ oligomers and fibrils.

*Aβ disaggregation assays*. Preformed Aβ aggregates were diluted with deionized water or EPPS solution (20 mM) and incubated for 1, 2, 3 and 7 days; final concentration of Aβ40 was 50 μM and that of Aβ42 was 25 μM. Aβ fibril formation was monitored using the ThT assay and transmission electron microscopy image analyses (Philips CM-30 transmission electron microscope)^60^. For ThT assay, preformed Aβ aggregates were incubated in 200, 20, 2, 0.2, 0.02, 0.002, 0.0002 or 0 mM of EPPS at 37 °C for 3 days. Fluorescence of Aβ-bound ThT was measured at 450 nm (ex) and 485 nm (em) using an EnSpire plate reader (Perkin-Elmer)^26^,^61^. To analyse size distribution of Aβ species after incubation, we employed SDS–PAGE and PICUP chemistry^29^. Briefly, Aβ samples were irradiated two times (each for 1 sec) to cross-link peptides with Ru(Bpy)(Cl_2_) and ammonium persulfate. Cross-linked Aβ samples were analysed on 1.0-mm-thick 10–20% gradient tris-tricine gels. After separation, gels were stained using silver-staining kit to visualize peptide bands. To confirm the cytotoxicity of Aβ aggregates, MTT (3-(4,5-dimethylthiazol-2-yl)-2,5-diphenyltetrazolium bromide) assays were performed according to the method described previously using HT-22 neuronal cell line (murine brain hippocampal cells)^13^. First, Aβ42 or Aβ40 was incubated for several days in 37 °C to obtain Aβ aggregates. Second, the aggregates were incubated for 7 days (Aβ(7d)) with or without EPPS. Finally, HT-22 cells were seeded and were exposed to the Aβ aggregates or Aβ(7d) for 24 hours. Final concentration of Aβ40 and Aβ42 for treatments was 2.5 and 5 μM with or without 2 mM EPPS in DMEM (0.05% DMSO). The MTT reagent and the stop solution were then added and absorbance was read via an EnSpire plate reader (Perkin-Elmer).

*Size exclusion chromatography*. SEC experiments were performed to confirm the disaggregated Aβ via EPPS^62^. We set the analysis system at 25 °C using a SEC-300 (Acclaim SEC-300, 5 μm, 4.6 × 300 mm, Thermo Scientific) column on an Ultimate 3000 UHPCL (Thermo Scientific). PBS was prepared as a mobile phase and the flow rate was set at 0.35 ml min^−1^. Ten microlitres of prepared Aβ42 samples and size controls (60 kDa BSA, 14 kDa thioredoxin and Aβ42 monomer) were injected and detected by ultraviolet absorbance at 280 nm.

*Surface plasmon resonance analysis*. The surface plasmon resonance analysis was performed using CM5 sensor chips and Biacore T200 instrument (GE Healthcare)^30^,^63^. Briefly, PBS-P (10 mM phosphate, 135 mM NaCl, 27 mM KCl and 0.05% SP20) was used as a buffer at 25 °C. Aβ40 was dissolved in DMSO to make 5 mM stock. The stock was tenfold diluted by deionized water and incubated at 37 °C for 5–7 days until the formation of oligomers, which was confirmed by SDS–PAGE with cross-link. Prepared Aβ40 oligomers were diluted with 10 mM sodium acetate solution (pH 4.0) to make 40 μg ml^−1^ of Aβ40 and immobilized to the sensor chip surface by amine coupling chemistry. The remaining activated surface groups were blocked with 1 M ethanolamine (pH 8.0); immobilization value was 9,000 RU and theoretical *R*_max_ was 525 RU. EPPS was prepared in the same buffer as serial-diluted samples: 0.075, 0.15, 0.3, 0.6, 1.2, 2.4, 4.8, 9.6 and 19.2 mM. Bimolecular interaction was measured by the binding response when EPPS was injected.

### Statistical analyses

Graphs were obtained with GraphPad Prism 5 and statistical analyses were performed with Student's paired *t*-tests, unpaired *t*-tests, repeated-measures analysis of variance or one-way analysis of variance followed by Bonferroni's *post-hoc* comparisons (*P<0.05, **P<0.01, ***P<0.001, ^#^*P*<0.05, ^##^*P*<0.01, ^###^*P*<0.001; other comparisons were not significant). The error bars represent the s.e.m.

## Additional information

**How to cite this article:** Kim, H. Y. *et al.* EPPS rescues hippocampus-dependent cognitive deficits in APP/PS1 mice by disaggregation of amyloid-β oligomers and plaques. *Nat. Commun.* 6:8997 doi: 10.1038/ncomms9997 (2015).

## Supplementary Material

Supplementary Information Supplementary Figures 1-9, Supplementary Tables 1-3, Supplementary Methods and Supplementary References.
