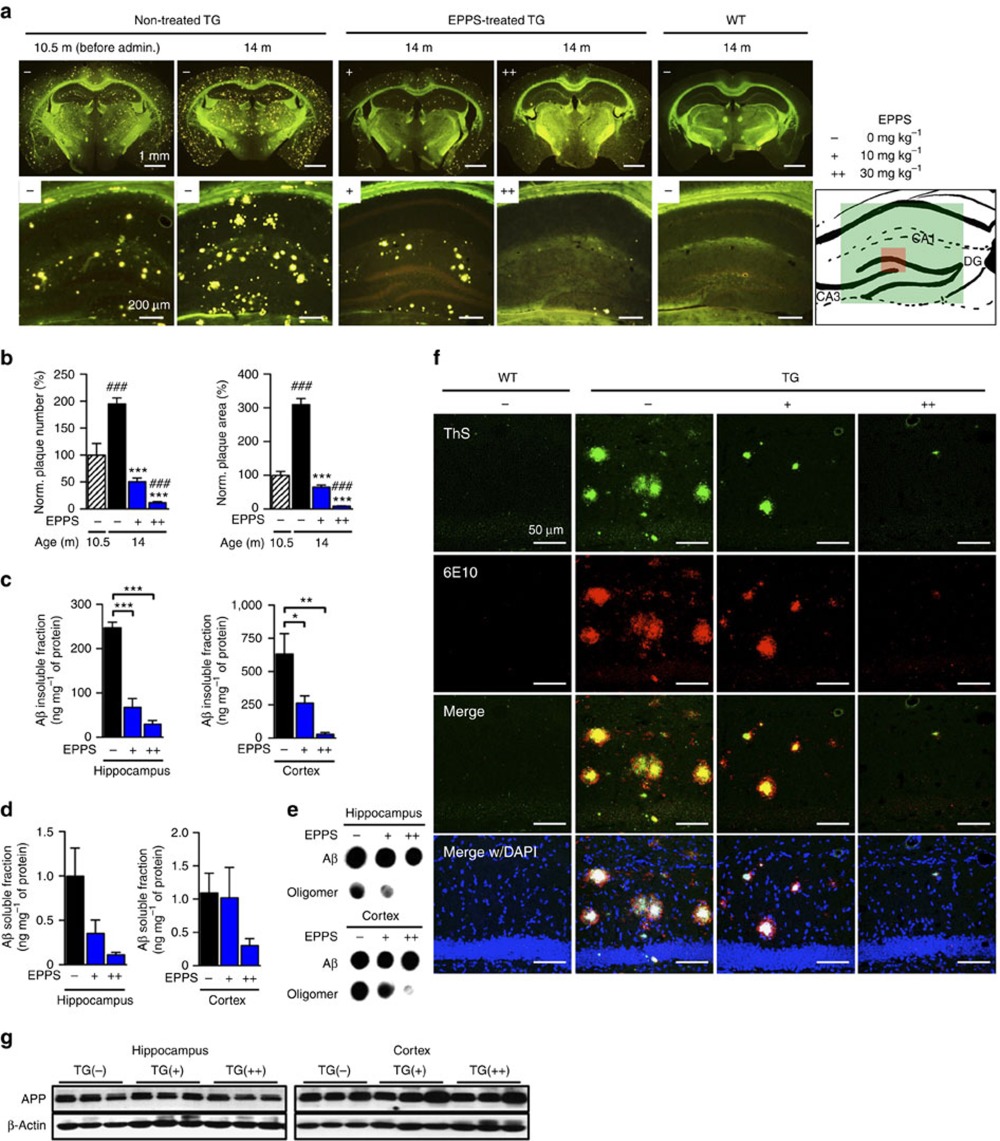# Corrigendum: EPPS rescues hippocampus-dependent cognitive deficits in APP/PS1 mice by disaggregation of amyloid-β oligomers and plaques

**DOI:** 10.1038/ncomms10755

**Published:** 2016-02-15

**Authors:** Hye Yun Kim, Hyunjin Vincent Kim, Seonmi Jo, C. Justin Lee, Seon Young Choi, Dong Jin Kim, YoungSoo Kim

*Nature Communications* 6: Article number: 8997 10.1038/ncomms9997 (2015); Published: 12082015; Updated: 02152016

This Article contains errors in Figs 3 and 4. In Fig. 3h, the *x* axis should have been labelled ‘TG', and in Fig. 4a, labels denoting the EPPS dose for each image were inadvertently removed. The correct versions of these two figures follow.

**Figure 3**


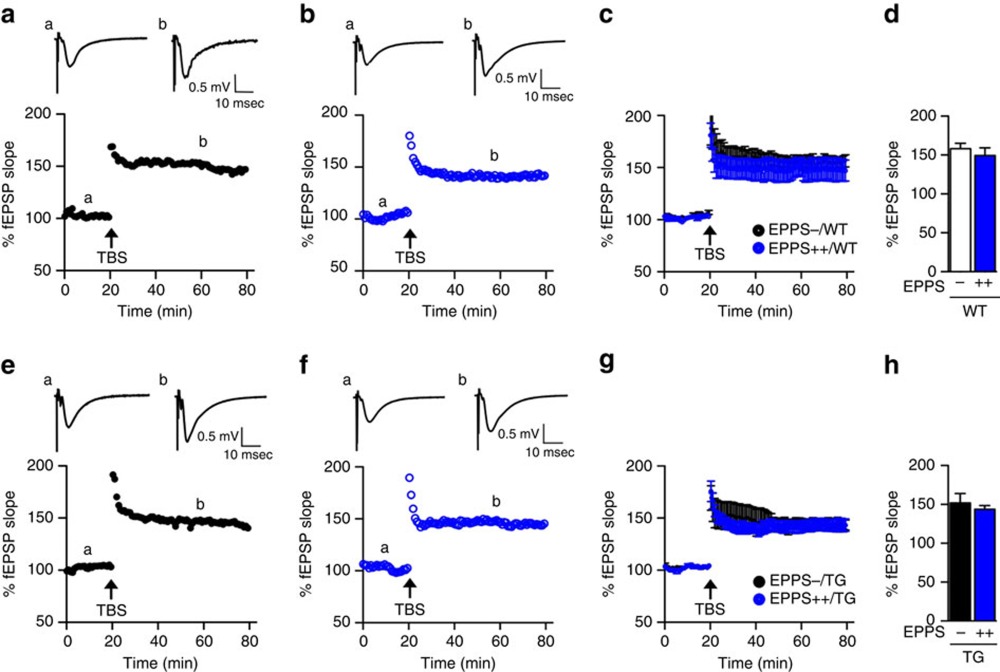


**Figure 4**