# Supplementary material for: EPPS rescues hippocampus-dependent cognitive deficits in APP/PS1 mice by disaggregation of amyloid-β oligomers and plaques
Source: Nat Commun. 2015 Dec 8;6:8997. doi: 10.1038/ncomms9997 (PMC4686862; doi:10.1038/ncomms9997)
Supplement: Supplementary Information — Supplementary Figures 1-9, Supplementary Tables 1-3, Supplementary Methods and Supplementary References. [file ncomms9997-s1.pdf]

Supplementary information

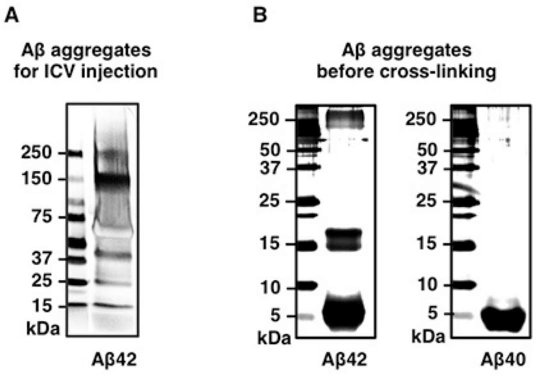

Supplementary Figure 1. SDS-PAGE with a silver-staining analyses of Aβ. A, SDS-PAGE with PICUP chemistry of prepared Aβ42 oligomers for intracerebroventricular (i.c.v.) injection. 10 μM Aβ42 in PBS (10% DMSO) was incubated at 37°C for 1 week. **B,** SDS-PAGE with non-cross-linked Aβ aggregates (Aβ42 = 25 μM, Aβ40 = 50 μM) as negative controls of PICUP chemistry[5] on Figure 5C and Supplementary Figure S5C.

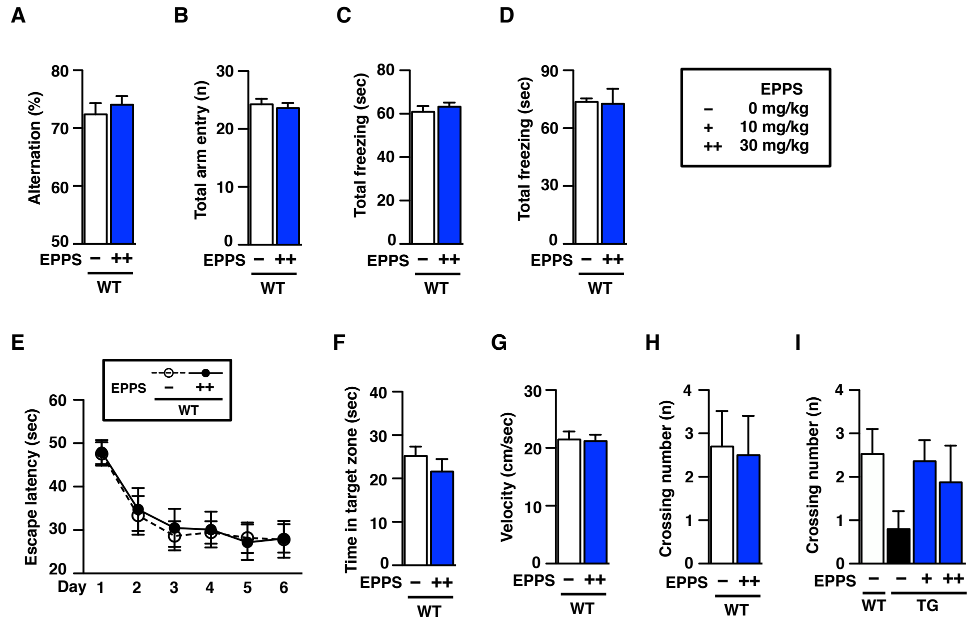

Supplementary Figure 2. EPPS does not affect behaviors in wild-type mice. A-H, EPPS 30 mg/kg/day (WT++, male, n = 10) was orally given to 10.5-month-old APP/PS1 for 3.5 months and their behavioral changes were compared to age-matched wild-types (WT−, male, n = 10). Y-maze, fear conditioning and Morris water-maze tests on 14-month-old APP/PS1 mice after EPPS administration of 3.5 month. (**A**) % alternation on Y-maze. (**B**) Total entry number into each arm of the Y-maze. (**C**) % total freezing from contextual fear-conditioning. (**D**) % total freezing in the cued task. (**E**) Hidden platform test and (**F**) probe test in Morris water maze. (**G**) Swim speeds of probe test and (**H**) crossing number of located hidden platform analysis. **I,** Crossing number of located hidden platform analysis of Morris water maze task of wild-type and transgenic mice. Error bars represent the SEMs. Student's *t*-test comparisons were performed in all statistical analyses of except escape latency and crossing number in Morris water maze (repeated-measures ANOVA and one-way ANOVA followed by Bonferroni's post-hoc comparisons, respectively) (*$P < 0.05$, **$P < 0.01$, ***$P < 0.001$; other comparisons were not significant).

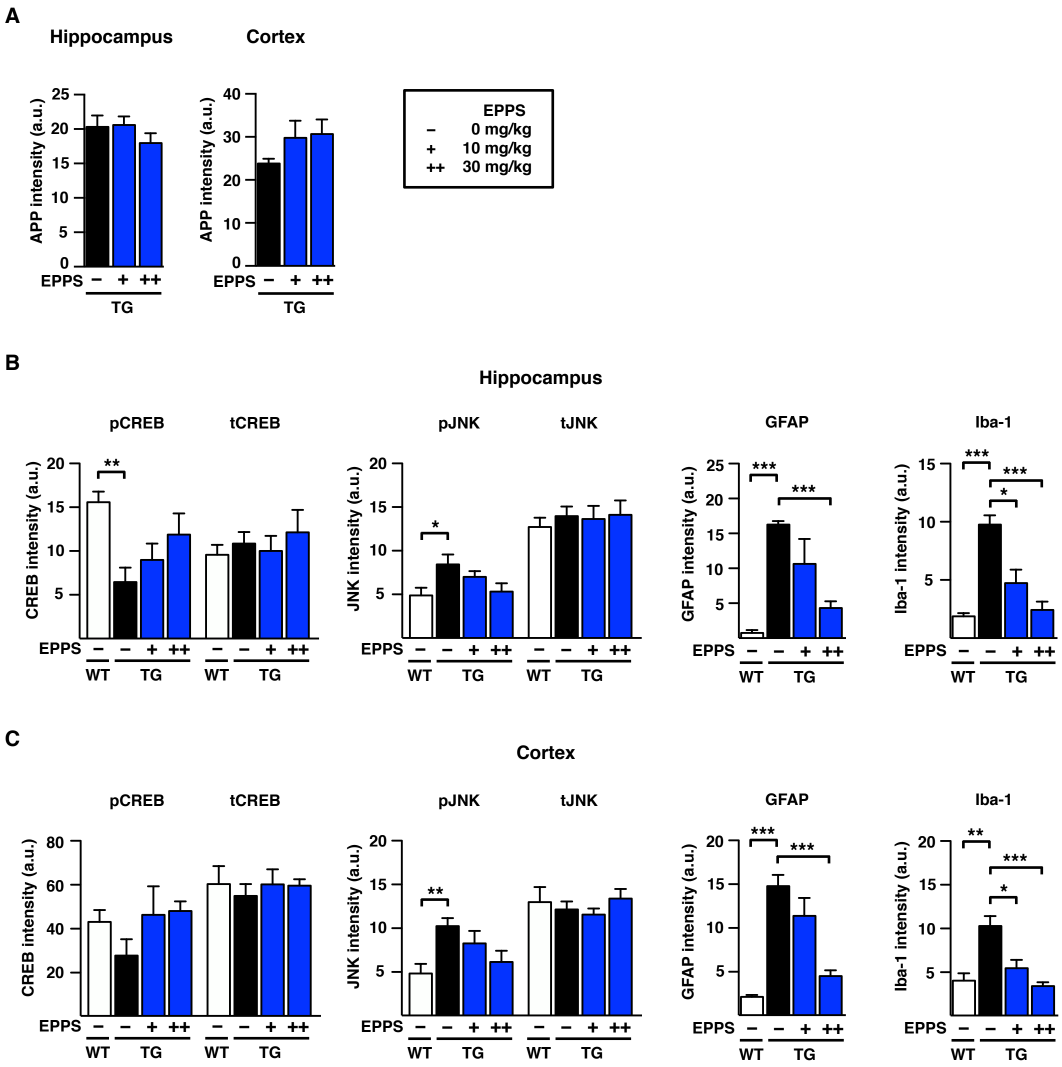

Supplementary Figure 3. Comparisons of APP expression level and plaque-dependent inflammation by treatment of EPPS. APP/PS1 mice and wild-types from the aforementioned behavioral tests were sacrificed and subjected to brain analyses. EPPS in 0 (TG−, male, n = 11), 10 (TG+, male, n = 11) or 30 mg/kg/day (TG++, male, n = 8) doses was orally given to 10.5-month-old APP/PS1 for 3.5 months and their brains were compared to age-matched wild-type brains. **A,** Densitometry analyses of APP expression level using hippocampal and cortical regions from Western blotting. **B,** Densitometry analyses of hippocampal proteins in Western blot. From the top, *P* values are 0.005 (pCREB) and 0.048 (pJNK), < 0.0001 and < 0.0001

(GFAP), 0.000, 0.000 and 0.014 (Iba-1). **C,** Densitometry analyses of cortical proteins from Western blotting. From the top, *P* values are 0.009 (pJNK), < 0.0001 and < 0.0001 (GFAP), 0.001, < 0.0001 and 0.014 (Iba-1). Error bars represent the SEMs. One-way ANOVA followed by Bonferroni's post-hoc comparisons tests were performed in all statistical analyses (*$P < 0.05$, **$P < 0.01$, ***$P < 0.001$; other comparisons were not significant).

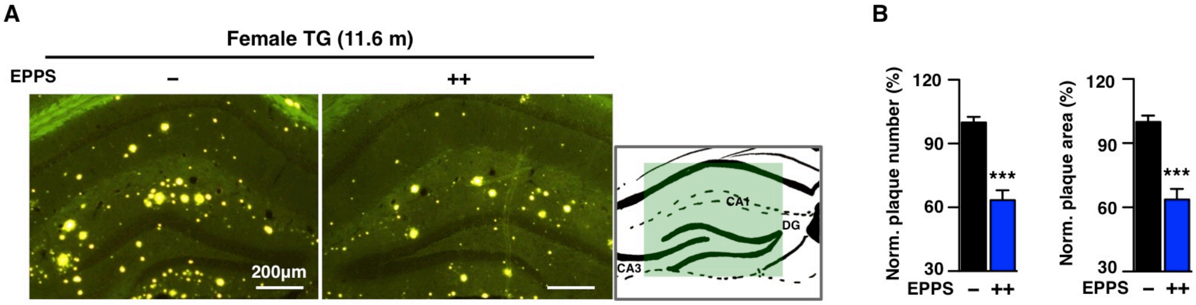

Supplementary Figure 4. EPPS disaggregates Aβ plaques in female mice. 10.6-month-old APP/PS1 transgenic mice were orally administered water (TG–, female, n = 6) or 30 mg/kg/day EPPS (TG++, female, n = 6) for 1 month (11.6-month-old). **A**, ThS stained-Aβ plaques in hippocampal regions of each group (scale bars, 200 μm) and **B**, normalized (%) number and area ($P < 0.0001$ for both) of plaques. ThS stained-Aβ plaques were significantly reduced in the hippocampal regions of EPPS TG++. The mouse brain schematic diagram was created by authors[4] (green box: region of brain imaging). Error bars represent the SEMs. Student's *t*-test was performed for all statistical analyses (*$P < 0.05$, **$P < 0.01$, ***$P < 0.001$; other comparisons were not significant).

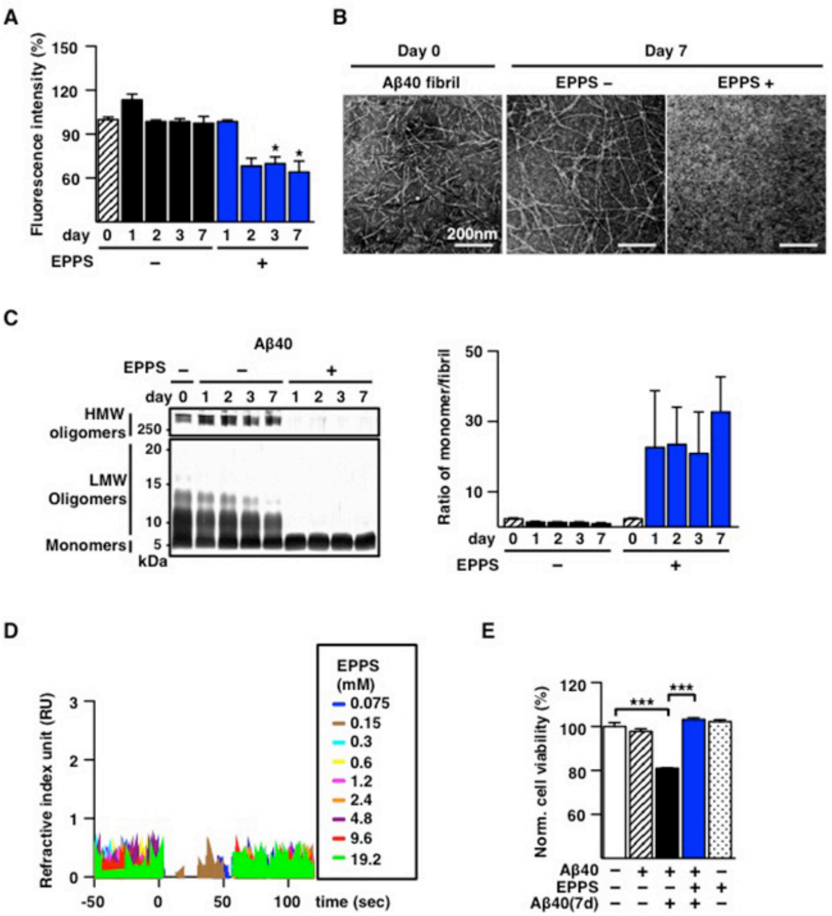

Supplementary Figure 5. EPPS disintegrates Aβ40 fibrils and oligomers into monomers. A-C, Pre-formed Aβ40 aggregates, fibrils and oligomers, were incubated with EPPS (for 1, 2, 3 and 7 days). (**A**) ThT assay. Aβ fibrils were gradually disassembled by EPPS. Fluorescence intensity was normalized to pre-formed Aβ aggregates (100%, Day 0). Statistical comparisons were made to day 0 (n = 4, Student's *t*-test; from the left: *P* = 0.033, 0.014, 0.037, 0.027). (**B**) Transmission electron microscopic images of EPPS-induced Aβ fibril disassembly. Scale bars, 200 nm. (**C**) The silver-staining for SDS-PAGE analysis of PICUP cross-linked Aβ aggregates and densitometry analysis in ratio of monomer to fibril (HMW: high molecular weight, LMW: low molecular weight). Oligomers and fibrils were disaggregated into monomers by EPPS. **D**, Surface plasmon resonance kinetics analysis. **E**, MTT assays. Aβ40: 5 μM Aβ40 aggregates,

Aβ40(7d): 5 μM Aβ40 aggregates were incubated for 7 days with/without EPPS (2 mM). Prepared samples were treated to HT-22 cells for 24 hr. Cell viability was normalized to that of non-treated cells (100%). All P values are < 0.0001 (n = 5). Error bars represent SEMs of independent triplicate measurements. One-way ANOVA followed by Bonferroni's post-hoc comparisons tests were performed in all statistical analyses. (*$P < 0.05$, **$P < 0.01$, ***$P < 0.001$; other comparisons were not significant).

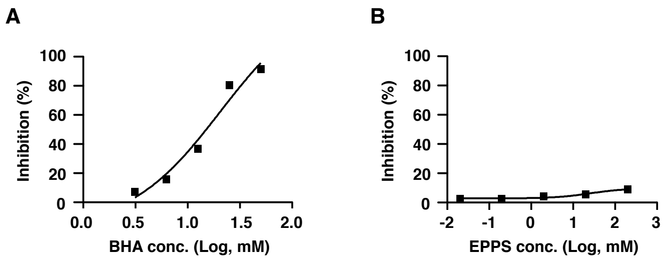

Supplementary Figure 6. DPPH radical scavenging test of EPPS. Antioxidant effect of EPPS was tested according to the 2,2-diphenyl-1-picrylhydrazyl (DPPH) radical scavenging activity test[3]. **A,** % inhibition of butylated hydroxyanisole (BHA) as a control. 50, 25, 12.5, 6.25 and 2.125 μg/mL concentrations were tested. IC_{50} value = 16.28 μg/mL. **B,** % inhibition of EPPS. 200, 20, 2, 0.2 and 0.02 mM concentrations were tested. All experiments had 9 replications.

Supplementary Figure 7. Full version of Western blots in Figure 4G.

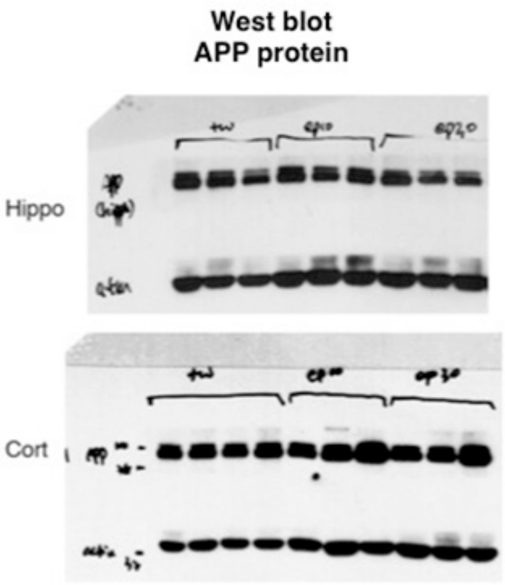

Supplementary Figure 8. Full version of Western blots in Figure 5A.

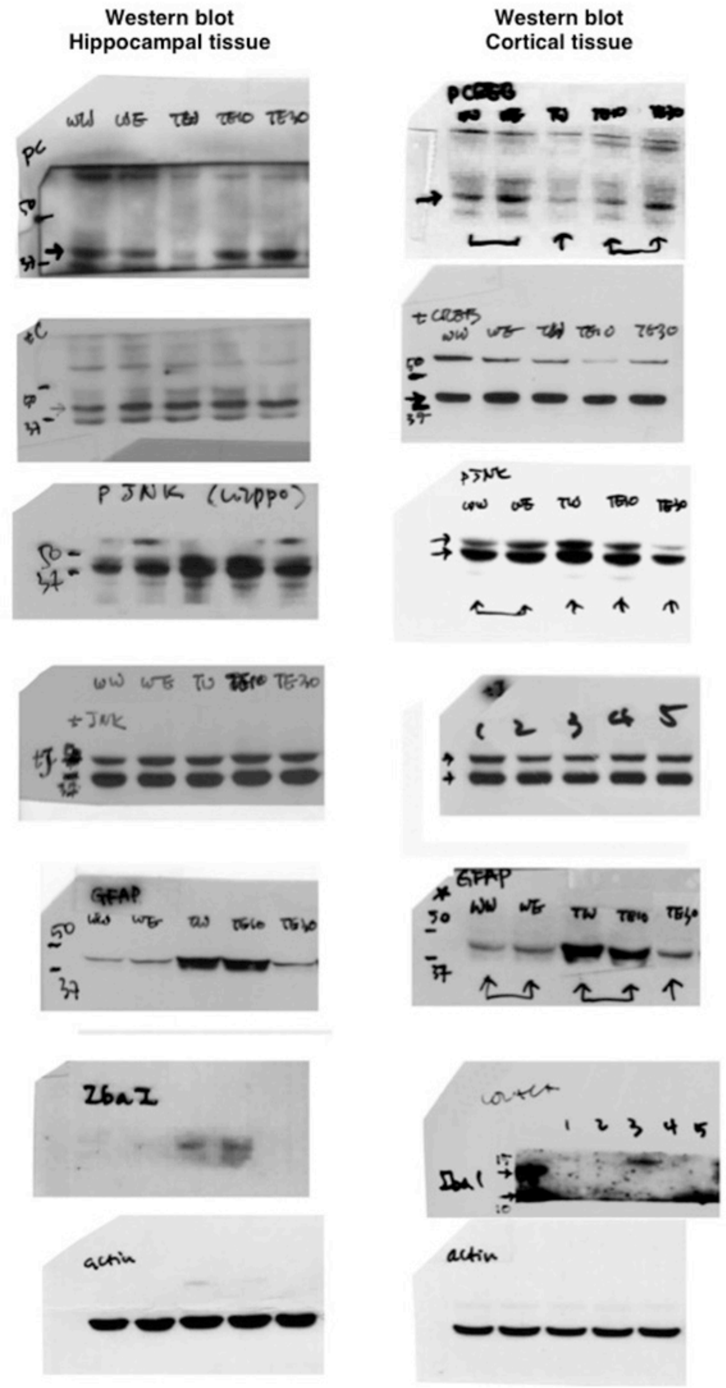

Supplementary Figure 9. Full length version of Silver stained gels in Figure 6C and Supplementay Figure 5C.

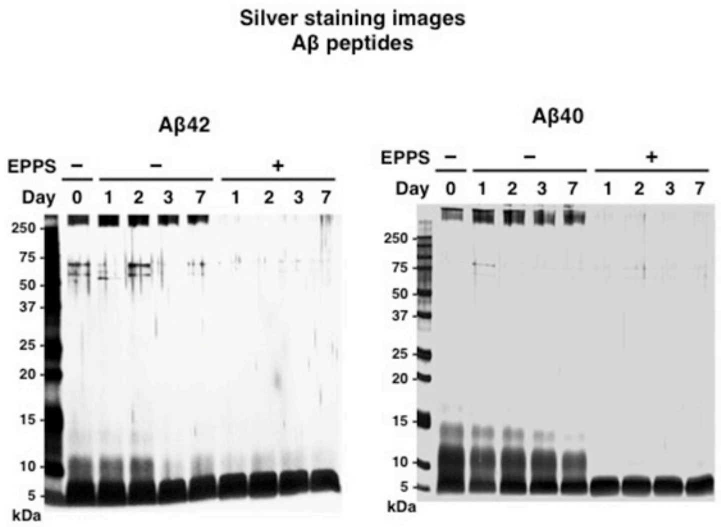

Supplementary Table 1. Plasma and brain concentration of EPPS in Sprague Dawley rats following oral administration in drinking water.

PO – 10 mg/kg/day

Time points (hr)	**Plasma concentration (ng/mL)** Animal number										
	201	202	203	204	205	206	207	208	209	Mean	SD
24	2.53	BLQ	2.47	BLQ	2.01	BLQ	BLQ	BLQ	2.78	**2.44**	**0.32**

Time points (hr)	**Plasma concentration (ng/mL)** Animal number: 201-209					Time points (hr)	**Brain concentration (ng/g)** Animal number: 201-209				
	G2-1	G2-2	G2-3	Mean	SD		G2-1	G2-2	G2-3	Mean	SD
72	4.21	8.38	8.41	**7.00**	**2.42**	72	10.79	6.22	5.55	**7.52**	**2.85**
120	3.81	2.99	4.30	**3.70**	**0.66**	120	8.01	4.92	8.16	**7.03**	**1.83**
168	3.29	3.11	6.50	**4.30**	**1.91**	168	4.94	11.38	6.23	**7.52**	**3.40**

PO – 100 mg/kg/day

Time points (hr)	**Plasma concentration (ng/mL)** Animal number										
	301	302	303	304	305	306	307	308	309	Mean	SD
24	33.44	7.96	35.30	13.35	31.98	10.67	19.67	41.96	19.25	**23.73**	**12.21**

Time points (hr)	**Plasma concentration (ng/mL)** Animal number: 301-309					Time points (hr)	**Brain concentration (ng/g)** Animal number: 301-309				
	G3-1	G3-2	G3-3	Mean	SD		G3-1	G3-2	G3-3	Mean	SD
72	84.82	55.16	98.48	**79.48**	**22.15**	72	11.74	10.29	31.55	**17.86**	**11.88**
120	82.03	36.60	70.71	**63.12**	**23.65**	120	9.64	6.11	11.70	**9.15**	**2.83**
168	37.24	27.53	36.44	**33.74**	**5.39**	168	12.29	11.42	10.24	**11.32**	**1.03**

LLOQ = 1.0 ng/mL for plasma and 5.0 ng/g for brain homogenate
NA: Not applicable.
BLQ: Below Limit of Quantitation

Supplementary Table 2. Brain/Plasma level of EPPS in Sprague Dawley rats following oral administration in drinking water.

Time points (hr)	PO – 10 mg/kg/day Concentration level (mL/g) Animal number: 201-209					Time points (hr)	PO – 100 mg/kg/day Concentration level (mL/g) Animal number: 301-309				
	G2-1	G2-2	G2-3	Mean	SD		G3-1	G3-2	G3-3	Mean	SD
72	2.564	0.742	0.660	**1.322**	**1.076**	72	0.138	0.187	0.320	**0.215**	**0.094**
120	2.099	1.644	1.899	**1.881**	**0.228**	120	0.118	0.167	0.166	**0.150**	**0.028**
168	1.504	3.658	0.959	**2.040**	**1.428**	168	0.330	0.415	0.281	**0.342**	**0.068**

NA: Not applicable.

Supplementary Table 3. Escape latency and significance (*P* value) of hidden platform test in Morris water maze.

Result from statistical analysis
Two-way repeated measures ANOVA<br>Genotype effect: F(1,46) = 47.196, *P* = 0.000<br>EPPS effect: F(2,46) = 10.232, *P* = 0.000
One-way repeated measures ANOVA<br>F(3, 46) = 17.627, *P* = 0.000<br>Bonferroni's post hoc analysis:<br>WT(−) vs. TG(−), *P* = 0.000<br>WT(−) vs. TG(+), *P* = 0.001<br>TG(−) vs. TG(++), *P* = 0.000<br>Other comparisons are not significant.
One-way ANOVA with Bonferroni's post hoc analysis (for each day)<br>Day 1: not significant<br>Day 2: WT(−) vs. TG(−), *P* = 0.005<br>Day 3: WT(−) vs. TG(−), *P* = 0.000<br>WT(−) vs. TG(+), *P* = 0.001<br>Day 4: WT(−) vs. TG(−), *P* = 0.000<br>WT(−) vs. TG(+), *P* = 0.037<br>TG(−) vs. TG(++), *P* = 0.008<br>Day 5: WT(−) vs. TG(−), *P* = 0.004<br>Day 6: WT(−) vs. TG(−), *P* = 0.000<br>TG(−) vs. TG(++), *P* = 0.019<br>Other comparisons are not significant.

(−): EPPS 0
(+): EPPS 10 mg/kg/day
(++): EPPS 30 mg/kg/day

Materials and Supplemental Methods

Materials 4-(2-hydroxyethyl)-1-piperazinepropanesulfonic acid (EPPS), dimethylsulfoxide (DMSO), thioflavin T (ThT), thioflavin S (ThS), tris(2,2'-bipyridyl)dichlororuthenium(II) (Ru(Bpy)(Cl_2)), ammonium persulfate, 2,2-diphenyl-1-picrylhydrazyl (DPPH) and butylated hydroxyanisole (BHA) were obtained from Sigma-Aldrich (St. Louis, MO). All Fmoc amino acids were from Novabiochem (San Diego, CA). Wang resin was obtained from RAPP Polymere GmbH (Baden-Wuerttemberg, Germany). Trifluoroacetic acid (Biograde) was from Halocarbon (River Edge, NJ). N,N-Dimethylformamide (BioAnalyzed) was from J.T. Baker (St. Louis, MO). 6-Cl-HOBt was from Peptide International (Louisville, KY). N,N-Diisopropylethylamine, N,N'-diisopropyl carbodiimide (DIC), piperidine, 1-ethyl-3-(3'-dimethylaminopropyl)carbodiimide (EDCI), and N-hydroxysuccinimide (NHS) were purchased from Aldrich (Milwaukee, WI). Deionized water was generated by a Milli-Q plus water purifier from Millipore (Bedford, MA). Disposable 0.20 μm sterile syringe filters and half-area black 96-well plates were purchased from Corning Corp. (Cambridge, MA and New York, NY). All reagents for cell culture and 3-(4,5-dimethylthiazol-2,5-diphenyl-tetrazolium bromide (MTT) were obtained from Gibco (Life Technologies Co.). All required agents and equipment for SDS-PAGE and blotting analyses were purchased from Bio-Rad (10-20% tris-tricine Criterion™ Precast Gel, tris/tricine/SDS buffer, 30% acrylamid/bis solution 29:1, protein assay dye) (Hercules, CA). To visualize peptide bands, PlusOne silver staining kit was employed from Amersham Biosciences (Piscataway, NJ). Insoluble fraction of Aβ in brains and Aβ in CSF quantifications used the Aβ42 human ELISA kit from Invitrogen (KHB3442 and KHB3544, respectively, Camarillo, CA). Antibodies for blotting and immunohistochemistry analyses are described in each method section.

Preparation of APP/PS1 double transgenic mice Double mutated transgenic mice were originally obtained from Jackson Laboratory (USA; strain name: B6C3-Tg (APPswe, PSEN1dE9) 85Dbo/J; stock number 004462), were supplied from Hanmi Pharm Co., Ltd. (Gyeonggi, Korea) and Medifron DBT (Gyeonggi, Korea). These APP/PS1 transgenic mice were maintained as double hemizygotes by crossing with wild-type mice on a B6C3F1 background strain. Both transgenes (*APP* and *PSEN1*) were doubly confirmed before and after whole animal experiments (PCR instrument from Bio-Rad, S1000 Thermal-Cycler) by the standard PCR condition providing Jackson Laboratory using PCR-premix and DNA extracted from mice tails (Cosmo-Genetech, G-taq PCR premix kit, CMT-6002). All mice (n = 4-5 per cage) were housed with free access to food and water, under controlled temperature conditions, and with a 12 hr:12 hr light:dark cycle. Behavioral tests were performed in the light period and in the same room where animals were being housed.

Wild-type mice behavioral studies To confirm EPPS effects on wild-type mice, 10.5-month-old C57BL6 wild-type male mice were prepared and administered EPPS (30 mg/kg/day) or water for 3 month. Y-maze, fear conditioning, and Morris water-maze were performed (n = 10 per group).

Western blot, dot blot, immunohistochemistry and Aβ plaque quantification analyses The primary antibodies used for Western blotting, dot blotting, and immunostaining were as follows: total-CREB (cAMP response element-binding protein, Santa Cruz sc-186, host: rabbit, 1:1,000), p-CREB (Cell Signaling #9198, host: rabbit, 1:1,000), Iba-1 (Millipore MABN92, host: mouse, 1:250 and Wako 019-10741, host: rabbit, 1:500), total-JNK (c-Jun NH2-terminal kinases, Cell Signaling #9252, host: rabbit, 1:1,000), p-JNK (Cell Signaling #9251, host: rabbit, 1:1,000),

GFAP (Millipore AB5541, host: chicken, 1:3000 and 1:500), 6E10 for Aβ and APP (Covance SIG-39300, host: mouse, 1:3,000 and 1:1,000), A11 for protein oligomer (Invitrogen AHB0052, host: rabbit, 1:500), GABA (Millipore AB175, host guinea pig, 1:1,000) and β-actin (a loading control) (Millipore MAB1501R, host: mouse, 1:5,000). Secondary antibodies (HRP-linked IgG) used were anti-mouse (sc-2005), anti-rabbit (sc-2030), and anti-chicken (sc-2901) from Santa Cruz. Fluorescent secondary antibodies (Alexa 555, Alexa 568 and Alexa 647) were purchased from Invitrogen or Jackson ImmunoResearch and used in 1:200 dilutions. Antibodies for sandwich-ELISA assays were used in the ELISA kit purchased from Invitrogen and performed according to the manufacturer's instructions.

Gender differences studies To confirm EPPS therapeutic effects of gender differences, 10.6-month-old APP/PS1 transgenic female mice were prepared and administered EPPS (30 mg/kg/day) or water for 1 month. Plaque quantification was performed as a same procedure of male tested groups (n = 6 per group).

Synthesis and purification of Aβ peptides Aβ40, H_2N-DAEFRHDSGYEVHHQKLVFFAEDVGSNKGAIIGLMVGGVV-COOH, and Aβ42, H_2N-DAEFRHDSGYEVHHQKLVFFAEDVGSNKGAIIGLMVGGVVIA-COOH, were prepared as previously described[1, 2]. In brief, both peptides were synthesized by stepwise solid phase peptide synthesis protocols for Fmoc (9-fluorenylmethoxycarbonyl) chemistry using DIC / HOBt. Wang resin was used for Aβ synthesis at a loading of 0.46 mmol OH/g (100-200 mesh). After Aβ synthetic reactions, RP-HPLC was performed by binary gradients of mobiles phase solution A and B, where A is 0.1% TFA (trifluoroacetic acid) in water and B is 0.09% TFA in acetonitrile. A

Vydac 214TP101522 column was employed for preparative RP-HPLC with detection at 220 nm during a linear gradient of 30-65%B over 30 min (10 mL/min flow rate).

DPPH radical scavenging activity Antioxidant effect of EPPS was tested according to the 2,2-diphenyl-1-picrylhydrazyl (DPPH) radical scavenging activity test[3]. Butylated hydroxyanisole (BHA), a well-known antioxidant, was used as a control and IC_{50} value was obtained as 16.28 µg/mL. BHA (50, 25, 12.5, 6.25 and 2.125 µg/mL) and EPPS (200, 20, 2, 0.2 and 0.02 mM) were tested with 9 replicated points. Absorbance values were recorded at 517 nm.

Statistical analyses Graphs were obtained with GraphPad Prism 5 and the statistic analyses were performed with Student's *t*-test comparisons, repeated-measures ANOVA or one-way ANOVA followed by Bonferroni's post-hoc comparisons ($^{*}P < 0.05$, $^{**}P < 0.01$, $^{***}P < 0.001$, $^{\#}P < 0.05$, $^{\#\#}P < 0.01$, $^{\#\#\#}P < 0.001$; other comparisons were not significant). Error bars represent the SEMs.

Supplementary References

1. Kim HY, Kim Y, Han G, Kim DJ. Regulation of in vitro Abeta1-40 Aggregation Mediated by Small Molecules. *J Alzheimers Dis* **22**, (2010).

2. Kim YS, Moss JA, Janda KD. Biological tuning of synthetic tactics in solid-phase synthesis: application to A beta(1-42). *J Org Chem* **69**, 7776-7778 (2004).

3. Liu JK, Hu L, Dong ZJ, Hu Q. DPPH radical scavenging activity of ten natural p-terphenyl derivatives obtained from three edible mushrooms indigenous to China. *Chem Biodivers* **1**, 601-605 (2004).

4. Paxinos G, Franklin BJ. *The Mouse Brain in Stereotaxic Coordinates*, 2nd edn. Academic Press (2001).

5. Bitan G, Teplow DB. Rapid photochemical cross-linking--a new tool for studies of metastable, amyloidogenic protein assemblies. *Acc Chem Res* **37**, 357-364 (2004).
